# Precision radiolabeled B-cell maturation nanobody for targeted PET imaging and radioligand therapy of disseminated multiple myeloma

**DOI:** 10.7150/thno.126920

**Published:** 2026-01-01

**Authors:** Behnaz Ghaemi, Colleen P. Olkowski, Falguni Basuli, Jianfeng Shi, Ryan Young, Dickran Kazandjian, Ola Landgren, Elizabeth Hill, Peter L. Choyke, Orit Jacobson

**Affiliations:** 1 Molecular Imaging Branch, Center for Cancer Research, National Cancer Institute, NIH, Bethesda, Maryland, 20892, USA.; 2 Chemistry and Synthesis Center, National Heart, Lung, and Blood Institute, NIH, Rockville, Maryland, 20850, USA.; 3 Lymphoid Malignancies Branch, Center for Cancer Research, National Cancer Institute, NIH, Bethesda, Maryland, 20892, USA.; 4 Sylvester Myeloma Institute, Sylvester Comprehensive Cancer Center, Department of Medicine, Myeloma Division University of Miami, Florida, 33146, USA.

**Keywords:** multiple myeloma, BCMA, nanobody, radioligand therapy, PET imaging, theranostics

## Abstract

**Background:** Multiple myeloma (MM) is an incurable plasma cell malignancy with limited disease-specific imaging options. Current diagnostic methods often fail to detect early disease states and minimal residual disease, highlighting the need for more precise molecular imaging and targeted therapeutic approaches. We developed a radiolabeled nanobody targeting B-cell maturation antigen (BCMA) to enable both high-contrast molecular imaging and targeted radioligand therapy in human MM models.

**Methods:** A high-affinity anti-BCMA nanobody was labeled with [^18^F]FPy-pyridine prosthetic group for PET imaging and [^131^I]I for radioligand therapy. Target expression and *in vitro* binding affinity and specificity were assessed using biolayer interferometry, flow cytometry, and cell-based assays. PET imaging studies were performed in subcutaneous MC38-human BCMA xenografts and systemic human MM models (H929 and RPMI8226 cell lines) administered intravenously in NSG mice. Therapeutic efficacy was evaluated using a fractionated treatment regimen with [^131^I]I-BCMA-Nb (four weekly injections of 7.4 or 18.5 MBq), monitoring tumor burden via bioluminescence imaging and [^18^F]FDG-PET. Toxicity assessment included body weight monitoring, complete blood counts, biochemical analyses, and histopathological examination.

**Results:** [^18^F]FPy-BCMA-Nb demonstrated high binding affinity and excellent tumor specificity with rapid systemic clearance. PET imaging showed significantly higher uptake in BCMA-positive lesions (6-8% ID/g) compared to controls (1% ID/g), with minimal kidney retention (<1% ID/g by 3 h). In systemic MM models, the tracer specifically targeted bone marrow lesions with high tumor-to-background ratios. Therapeutic studies revealed dose-dependent tumor regression, with the 18.5 MBq [^131^I]I-BCMA-Nb regimen achieving 100% complete remission in treated mice. Biochemical and histopathological analyses confirmed minimal systemic toxicity, restoration of normal hematopoiesis, and significant reduction in BCMA expression and proliferation markers post-treatment.

**Conclusion:** This BCMA-targeted nanobody platform offers a promising theranostic approach for precise detection and treatment of disseminated multiple myeloma. The combination of exceptional tumor specificity, minimal off-target accumulation, rapid clearance, and potent therapeutic efficacy, along with a favorable safety profile, supports its potential for clinical translation in MM diagnosis and therapy.

## Introduction

Multiple myeloma (MM) is a relatively common hematologic malignancy, accounting for approximately 10% of all blood cancers 1, 2. The disease is highly heterogeneous, with complex underlying genetic abnormalities, clinical presentations, and variable responses to treatment. Despite significant therapeutic advances, MM remains an incurable disease. Most patients ultimately relapse, even after initially achieving deep responses, highlighting the urgent need for more sophisticated and precise diagnostic and monitoring approaches 3-5.

A fundamental diagnostic challenge in MM stems from the limitations of current detection methods. Traditional diagnostic techniques including bone marrow biopsy, serum protein electrophoresis, and conventional imaging modalities like X-rays, CT, and standard [^18^F]FDG-PET often fail to detect precursor states such as monoclonal gammopathy of undetermined significance (MGUS) and the smoldering stage of multiple myeloma 2. These precursor states represent critical points in disease progression where early intervention could potentially prevent advancement to symptomatic MM. However, the lack of sensitivity of current imaging methods typically means these early states of disease remain undetected, significantly reducing the likelihood of successful treatment 6, 7. Furthermore, monitoring treatment response remains challenging with conventional imaging, which lacks sensitivity for small or heterogeneous lesions. As a result, residual or therapy-resistant malignant cells may remain undetected, ultimately contributing to disease recurrence. For instance, currently it is difficult to detect small populations of resistant malignant cells that persist after therapy and ultimately lead to disease recurrence. This limitation undermines the effectiveness of even the most advanced treatment regimens, as clinicians cannot accurately identify patients who require additional or alternative therapies despite appearing to be in remission by standard assessments 6-8. Positron emission tomography (PET) has emerged as a highly sensitive modality for detecting small amounts of disease with specificity based on tumor-targeted PET agents.

Among the various molecular targets identified for MM, B-cell maturation antigen (BCMA) has emerged as an ideal candidate. BCMA is characterized by its uniform and sustained expression on malignant plasma cells across the entire spectrum of disease progression, from MGUS to relapsed/refractory MM, with minimal to no expression in healthy tissues outside the B-cell lineage 9, 10. This selective expression pattern makes BCMA an excellent target for both diagnostic imaging and targeted therapy, potentially allowing for earlier detection of disease, more accurate staging, and precise monitoring of treatment response 9, 10.

Previous attempts at PET imaging based on anti-BCMA antibodies labeled with the long half-life PET isotope [^89^Zr]Zr (T_1/2_ of 78.4 h) were limited by the large size (150 k Da) and slow clearance of the conjugate from the blood. This prolonged circulation time reduces target to background ratios, thus masking bone lesions and minimal residual disease (MRD) detection, while requiring extended imaging periods (multiple days) to allow for adequate clearance 11. Furthermore, labeling antibodies with long-lived radiotherapeutic isotopes (such as [^177^Lu]Lu, [^90^Y]Y, or [^225^Ac]Ac) might result in toxicity due to their extended circulation time in the bloodstream, potentially delivering prolonged exposures of radiation to healthy tissues and compromising the therapeutic window.

Smaller targeting agents such as single-domain antibody fragments (nanobodies) overcome the problem of prolonged clearance 12, 13. Derived from camelid heavy-chain antibodies, nanobodies offer significant advantages over antibodies including enhanced tumor penetration due to their small size (~15 k Da), quicker clearance from non-target tissues, reduced off-target accumulation, and improved image contrast. Their advantageous pharmacokinetic profile allows imaging with short-lived PET isotopes such as [^68^Ga]Ga or [^18^F]F (~ 68 and 110 minutes half-life respectively), enabling same-day imaging protocols that are more convenient for patients and more efficient for clinical workflow. Additionally, their rapid clearance from the bloodstream substantially minimizes systemic toxicity during radioligand therapy, potentially allowing for higher radiation doses to be delivered.

In this study, we describe the synthesis of the [^18^F]FPy-BCMA nanobody radiotracer, characterize its properties *in vitro*, evaluate its stability, and demonstrate its effectiveness for PET imaging across multiple mouse models. Additionally, we explore its potential therapeutic application through [^131^I]I labeling, monitoring both tumor growth suppression and associated toxicity profiles. Although the current study focuses on systemic MM imaging and radionuclide therapy, the pharmacokinetic properties of nanobodies, rapid blood clearance, high target specificity, and excellent tumor-to-background contrast, make them well-suited for future applications requiring extreme sensitivity, such as MRD detection or early-stage disease assessment (e.g., MGUS and smoldering myeloma). While these applications were not tested here, the development of highly specific BCMA-targeted PET tracers establishes the foundational framework for future investigations aimed at improving detection of minimal disease burden and early precursor states.

## Materials and Methods

### Radiolabeling and Serum Stability

The BCMA-specific nanobody was obtained from Creative Biolabs (Cat. No. VS-0923-YJ96) and consists of a camelid anti-human BCMA single-domain antibody (VHH). [^18^F]F was obtained from the NIH clinical center cyclotron unit and [^131^I]I was purchased from International Isotopes Inc. [^18^F]FDG was purchased from Cardinal Health. [^18^F]FPy-TFP was conjugated to the nanobody and purified according to published protocol 14. [^131^I]I-BCMA-Nb was prepared using the Iodogen tube (ThermoFisher) method using 15-17 nmol of BCMA-Nb and 15-20 mCi of [^131^I]I for 15 minutes at RT, with a total reaction volume of 300 µL PBS. Purification of the labeled nanobody was done using PD-10 columns with PBS as the eluent. Quality control and purity assessment of labeled nanobody was done using HPLC with a size exclusion column (Tosoh Bioscience LLC, TSKgel SuperSW3000, 4.6 mm ID x 30 cm, 4µm) and eluent consisting of 0.1 M sodium phosphate, 0.1 M sodium sulfate, 0.05% sodium azide, and 10% isopropyl alcohol (pH 6.8) at a flow rate of 0.35 mL/min. Radioactive thin-layer chromatography (radioTLC) was done on Eckert & Ziegler TLC scanner (B-AR2000) using iTLC-SG plates (ThermoFisher) and 0.1 M citrate buffer (pH 5). *In vitro* serum stability was evaluated by incubating approximately 1 mCi of [^18^F]FPy-BCMA or 0.1 mCi of [^131^I]I-BCMA-Nb in mouse and human serum (Sigma Aldrich) at 37 °C for up to 3 h, with aliquots analyzed at 1, 2, and 3 h using the HPLC and radioTLC conditions described above.

### Cell Culture

Wild-type MC38 (MC38-WT, RRID: CVCL_B288) cells were obtained from ATCC, while all other cell lines were purchased from Kyinno Biotechnology Inc. MC38-WT cells were cultured in DMEM medium, and all other cell lines were cultured in RPMI-1640. All cultures were supplemented with 10% fetal bovine serum and maintained in a humidified incubator at 37 °C with 5% CO_2_.

### Flow Cytometry

The cells were analyzed for BCMA expression using flow cytometry with an Alexa-647-conjugated anti-human BCMA antibody (RRID: AB_2687258) or isotype control (BioLegend, RRID: AB_2864287). A 2% bovine serum solution was used as the assay buffer. A total of 1 × 10⁶ cells/mL were resuspended in a blocking buffer containing 2% bovine serum albumin, 2% rat serum, and 2% human serum dissolved in the assay buffer. Anti-human BCMA antibody or isotype control (1:50 dilution) was added to each cell line and incubated for 1 h at RT. The cells were then washed three times with the assay buffer. Propidium iodide was used to stain dead cells. Fluorescence was analyzed using a CytoFLEX flow cytometer (BD Biosciences), and the results were processed using FlowJo software (RRID:SCR_008520).

### Kinetic Binding Assay by Biolayer Interferometry

Biolayer interferometry studies were conducted similarly to the published procedure using 1ug/mL of biotinylated human BCMA protein (ACROBiosystems, BCA-H82E4) and 100nM concentration of BCMA-Nb in PBS as an eluent 14.

### Saturation Binding Assays

A saturation binding assay was conducted using a membrane 96-well plate (Sigma Aldrich) with increasing concentrations (0.01-200 nM) of [^18^F]FPy-BCMA-Nb, using MC38-BCMA, H929 (RRID: CVCL_1600), and RPMI8226 (RRID: CVCL_0014) cells (50,000 cells per well). To block nonspecific binding, a 100-fold excess of BCMA-Nb was used. The assay was performed for 1 h at RT, followed by three washes with PBS. The membrane was then dried on a heater for 15 minutes. Samples were removed from the plates and read using a gamma counter (Revvity). Each concentration was tested in triplicate. The data were analyzed using GraphPad Prism software.

### Cell Uptake, Internalization and Efflux

MC38-WT and MC38-BCMA cells (100,000 cells per well) were seeded into 24-well plate 24 h prior to the study. Suspended H929 cells were counted and placed into Eppendorf tubes on the day of the study. All assays were performed using FBS-free medium. 74 KBq of [^18^F]FPy-BCMA-Nb in 0.5mL of medium was added to each well/Eppendorf tube, and incubated at 37°C. At each time point, the medium was removed either by suction or centrifugation, and the cells were washed twice with PBS. For uptake studies, cells were lysed using 0.1M NaOH. For internalization studies, cells were incubated with 0.5 mL of acidic buffer (50 mM glycine, 100 mM NaCl, pH 2.8) for 1 minute, then centrifuged and washed twice with PBS. Finally, 0.1M NaOH was added. For cell uptake and efflux using [^131^I]I-BCMA-Nb, the same procedure was used, but for the efflux studies, [^131^I]I-BCMA-Nb was added to H929 cells in Eppendorf tubes and incubated for 3 h at 37°C. Then, cells were washed twice with PBS, and incubated with RPMI-1640 medium for 5, 30, 60, 120 and 180 min at 37°C. After twice washing with PBS, cells were harvested by addition of 0.1 M NaOH. All cell suspensions in NaOH were collected and measured in a gamma counter. Data was analyzed using GraphPad Prism software. Each time point was performed in triplicate.

### Mouse Xenograft Models

All animal experiments were carried out in accordance with protocol MIP-006 approved by the NCI animal care and use committee (ACUC). Athymic nude and NSG mice were purchased from the Jackson Laboratory. For subcutaneous MM tumors, 1 × 10⁶ MC38-BCMA and MC38-WT cells were resuspended in sterile PBS mixed with Matrigel matrix (Corning) at a 1:1 ratio and implanted into the right and left posterior flanks of female athymic nude mice (7-8 weeks old, RRID: IMSR_JAX:002019). Tumors were allowed to grow until they reached a volume of 300-350 mm³ before imaging. For the systemic MM model, 1 × 10⁶ H929 or RPMI8226 cells were injected intravenously into 7-8-week-old NSG female mice in a volume of 200 µL sterile PBS. Bioluminescence imaging confirmed sufficient tumor burden 20-21 days after cell injection.

### PET and Biodistribution Studies

Mice were injected intravenously with 3.7 MBq (10-15 µg of BCMA-Nb) of [^18^F]FPy-BCMA-Nb (n = 6 mice per group), in sterile PBS, and scans were obtained at 30 min, 1, 2, and 3 h post injection using a MR Solutions PET/CT scanner and acquisition time of 5-15 min. Blocking studies were done by co-injection of [^18^F]FPy-BCMA-Nb with unlabeled BCMA-Nb (50-fold excess). Biodistribution was carried out after the last PET/CT scan time point. Mice were euthanized, and the tumor, heart, lung, liver, spleen, stomach, intestine, pancreas, kidneys, muscle, bone, and blood were harvested and weighed. The amount of radioactivity was determined by a gamma counter. Results are expressed as percentages of the injected dose per gram of tissue (% ID/g).

For the [^18^F]FDG studies, 3.7 MBq of [^18^F]FDG was administered intravenously to mice that were anesthetized for 30 minutes prior to imaging to reduce non-specific glucose uptake by muscles (n = 6 mice per group). The mice remained under anesthesia for an additional h before being scanned for 10 minutes using a Mediso PET/CT scanner. All PET images were reconstructed using the ordered-subset expectation maximization (OSEM) algorithm, and image analysis was conducted with MIM software Inc (RRID: SCR_006437). Statistical analysis was performed using GraphPad Prism (Version 10, RRID: SCR_002798).

### Targeted Radiotherapy Studies of MM Using [^131^I]I-BCMA Nb and Single-Photon Emission Computed Tomography (SPECT)

Targeted radiotherapy studies were conducted in H929 systemic MM model. Bone lesions in the spine and femur were confirmed using BLI. Mice were randomized into four treatment cohorts: PBS (n = 8), 7.4 MBq of [^131^I]I-BCMA-Nb (n = 8), 18.5 MBq of [^131^I]I-BCMA-Nb (n = 8), and 18.5 MBq of [^131^I]I-non-specific-Nb (n = 5). Therapeutic activities of 7.4 MBq and 18.5 MBq were selected based on prior studies demonstrating safe and effective [^131^I]I-nanobody therapy in mice and supported by the rapid clearance kinetics observed for the BCMA nanobody 15, 16. The lower activity represented a conservative dose, while the higher activity approached the upper bound of tolerated [^131^I]I exposure under a fractionated weekly regimen.

Mice received intravenous injections once per week for four weeks, and therapeutic response was assessed by monitoring body weight, survival rate, and complete blood count (CBC) before and after each injection until the end of the study. Endpoint criteria, as defined by the NCI ACUC, included >15% body weight loss, paralysis or severe weakness, or abnormal behavior indicative of pain or distress. These criteria were also used for Kaplan-Meier survival analysis. The time from treatment initiation to meeting any endpoint criterion was recorded as the survival time. These data were used to construct Kaplan-Meier survival curves, with animals still alive at study completion or removed for reasons unrelated to tumor burden treated as censored observations. Statistical differences between groups were determined using the log-rank (Mantel-Cox) test and confirmed by the Gehan-Breslow-Wilcoxon test in GraphPad Prism.

SPECT imaging was performed 3 h after administering 18.5 MBq [^131^I]-BCMA-Nb in a representative mouse (n = 2) using a Bioscan SPECT/CT scanner. The CT acquisition time was 5 minutes, and the SPECT acquisition lasted for 40 minutes. Image reconstruction was performed using the manufacturer's software, and the images were then exported to MIM for further analysis.

### Bioluminescence Imaging (BLI)

BLI was performed before and after each treatment regimen. Mice were injected intraperitoneally (i.p.) with 100 μL of D-luciferin (15 mg/mL, GoldBio) and anesthetized using 2% isoflurane. Imaging was conducted using an IVIS Spectrum (Revvity) system, and luminescence signals were acquired 10-15 minutes post-luciferin injection. Total flux (photons/sec) was quantified from regions of interest (ROI) using Living Image software (Revvity, RRID:SCR_014247). Normalization was performed against baseline signal prior to treatment.

### Bone Collection, Fixation, Decalcification, and Cryo-Sectioning

Femurs and spines were collected from each treatment group both before and after therapy. Immediately upon dissection, tissues were placed in Z-FIX (Anatec Ltd.) at RT for 48 h. The fixed bones were then transferred to 0.5 M EDTA (pH 7.4) for 10 days to achieve complete decalcification, with regular changes of the decalcification solution. Afterward, the bones were briefly rinsed in PBS, embedded in a cryoprotectant medium (OCT, Tissue-Tek), and frozen. Finally, 10 μm-thick sections were cut using a cryomicrotome (Leica CM3050S) and mounted onto glass slides. All slides were stored at -20 °C for subsequent histological or immunohistochemical analyses.

### Immunohistochemical Staining

Immunohistochemical staining of BCMA was performed on 10 μm bone cryosections thawed at RT. Sections were rinsed in PBS and incubated with 3% hydrogen peroxide for 10 minutes, followed by a blocking step in 5% normal goat serum (Cell Signaling Technology) for 1 h at RT. Anti-BCMA primary antibody (Abcam, RRID:AB_2932172) was diluted 1:250 in antibody diluent (Cell Signaling Technology) and applied overnight at 4 °C. After thorough PBS washes, sections were incubated with the secondary antibody (HRP, rabbit, RRID: AB_10544930), followed by DAB substrate solution (Cell Signaling Technology), and then counterstained with hematoxylin (Cell Signaling Technology).

### Immunofluorescent Staining (Ki-67)

For Ki-67 staining, separate 10 μm bone cryosections were thawed at RT, rinsed in PBS, and permeabilized with 0.1% Triton X-100 for 10 minutes. After blocking in 5% normal goat serum for 1 h at RT, sections were incubated overnight at 4 °C with a FITC-conjugated Ki-67 monoclonal antibody (Invitrogen, AB_10687464), diluted 1:250. Excess antibody was removed by washing with PBS, and nuclei were counterstained with DAPI (ThermoFisher).

### Immunofluorescent Staining (CD45)

Femurs from control mice and mice treated with 18.5 MBq [^131^I]I-BCMA-Nb were processed as described above, including fixation and decalcification prior to cryosectioning. Sections were equilibrated in PBS and incubated in 5% goat serum for 1 h at room temperature to block nonspecific binding. Slides were then incubated overnight at 4 °C with Alexa Fluor 488-conjugated anti-human CD45 antibody (BioLegend, RRID: AB_2721364) at a 1:100 dilution. After primary antibody incubation, sections were washed extensively in PBS and counterstained with DAPI to visualize nuclei. Slides were mounted using an aqueous antifade mounting medium and imaged using a scanning microscope (VS200, Olympus).

### Immunofluorescence Imaging and Quantification

Slides were then rinsed, dehydrated through graded alcohols, cleared in xylene for 10 minutes, and mounted using a permanent medium (Cell Signaling Technology). Images were captured using a scanning microscope (VS200, Olympus). The number of Ki-67-positive nuclei was quantified by manual counting across 10 fields of view per slide, with three slides per mouse and three mice per group. Image analysis and quantification were performed using ImageJ (NIH, RRID:SCR_003070).

### Complete Blood Count

Blood samples were collected before and after treatment regimen through the cheek puncture and kept in EDTA-treated tubes (Microvette® 100). Blood count tests were carried out with a hematology analyzer (PLA1000, Pro-Lab Diagnostics) following the vendor's protocol.

### Liver and Kidney Toxicities

Following the treatment regimen, blood samples were collected via cardiac puncture into EDTA-treated Microvette® tubes. A 150 µL aliquot of blood was placed onto a quantitative analysis disk (ABAXIS) and processed using a VetScan VS2 blood analyzer. To assess liver function, serum levels of alkaline phosphatase (ALP), alanine aminotransferase (ALT), globulins, and total bilirubin were measured. To evaluate kidney function, total protein, albumin, blood urea nitrogen (BUN), potassium (K), and sodium (Na) were analyzed. All biochemical assays were conducted using standard clinical chemistry methods. Data were analyzed in GraphPad Prism and compared to reference values for 10-week-old female NSG mice.

### Post-Mortem Analysis of Vital Organs

Following euthanasia, liver and kidney tissues were harvested from treated and control mice for post-mortem histological analysis. The organs were fixed in 4% Paraformaldehyde for 24 h, followed by 72 h incubation in 30% sucrose. Tissue sections (5-10 µm thick) were prepared using a microtome (Leica CM3050S), mounted on glass slides, and stained with hematoxylin and eosin following standard histological procedures. The slides were examined using scanning microscope (VS200, Olympus). The severity of any observed lesions was compared between treated and control groups to assess potential treatment-induced toxicity.

### Statistical Analysis

Statistical analysis was performed using GraphPad Prism software. Data are presented as mean ± standard deviation, as indicated in the figures. Two-tailed paired and unpaired Student's t-tests were used to assess differences within groups and between groups, respectively. Additionally, one-way and two-way ANOVA were conducted for multiple group comparisons. Statistical significance was defined as follows: P < 0.05 (*), P < 0.01 (**), P < 0.001 (***).

## Results

### Radiolabeling and Stability of Nanobody

**Figure 1:**
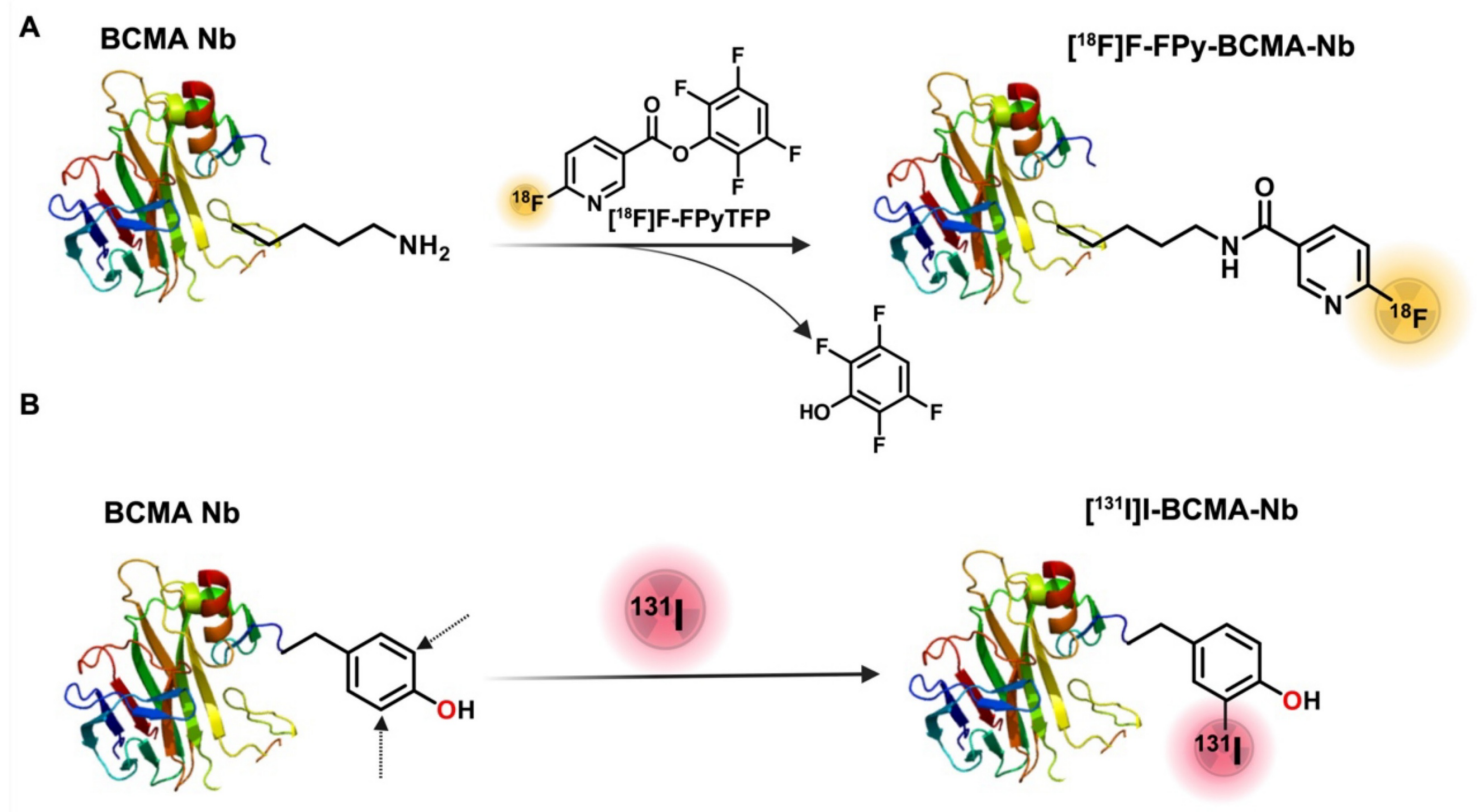
Schematic of BCMA-Nb radiolabeling with (A) [^18^F]FPy and (B) [^131^I]I.

The synthesis of [^18^F]FPy-BCMA-Nb followed a two-step process (Figure 1A), according to our recent protocol 14, 17. Briefly, 6-fluoronicotinic acid-2,3,5,6-tetrafluorophenyl ester ([^18^F]FPy-TFP) was prepared on a Sep-pak at room temperature (RT), and without further purification, it was reacted with BCMA-Nb, purified and reformulation in Phosphate-buffered saline (PBS). The final product, [^18^F]FPy-BCMA-Nb, exhibited a radiochemical purity (RCP) of > 99% with no significant aggregation, as confirmed by high-performance liquid chromatography (HPLC) analysis (Figure S1A). The specific activity (SA) and overall radiochemical yield (RCY) based on [^18^F]F initial activity were determined to be 100-300 Ci/mmol and 12-18% (decay-corrected, 45 minutes synthesis time), respectively (n = 9). Stability studies in mouse and human serum were conducted using HPLC up to 3 h post-incubation (Supplemental Figure S1B-E), revealing excellent stability (> 95%) with no detectable metabolite formation.

To assess the potential of BCMA-Nb for MM radionuclide-targeted therapy, we used [^131^I]I direct labeling on the tyrosine residues of the nanobody (Figure 1B). The labeling was efficiently done in Iodogen tubes and resulted in high RCP of the labeled nanobody (> 99%), RCY of 80-82% (not decay-corrected), and a specific activity of 1500-1600 Ci/mmol. Similar to [^18^]FPy-BCMA-Nb, the [^131^]I-BCMA-Nb demonstrated high stability in PBS and serum up to 3 h post-incubation. The proportion of intact labeled nanobody decreased only slightly from >99% at time 0 (retention time 9.9 min) to 95.2% at 3 h, accompanied by the appearance of two minor radiochemical peaks at 5.6 min (2.5%) and 15 min (2.2%) (Supplemental Figure S2).

### Superior Tumor Targeting with Reduced Renal Uptake of [^18^F]FPy-BCMA Nb

**Figure 2:**
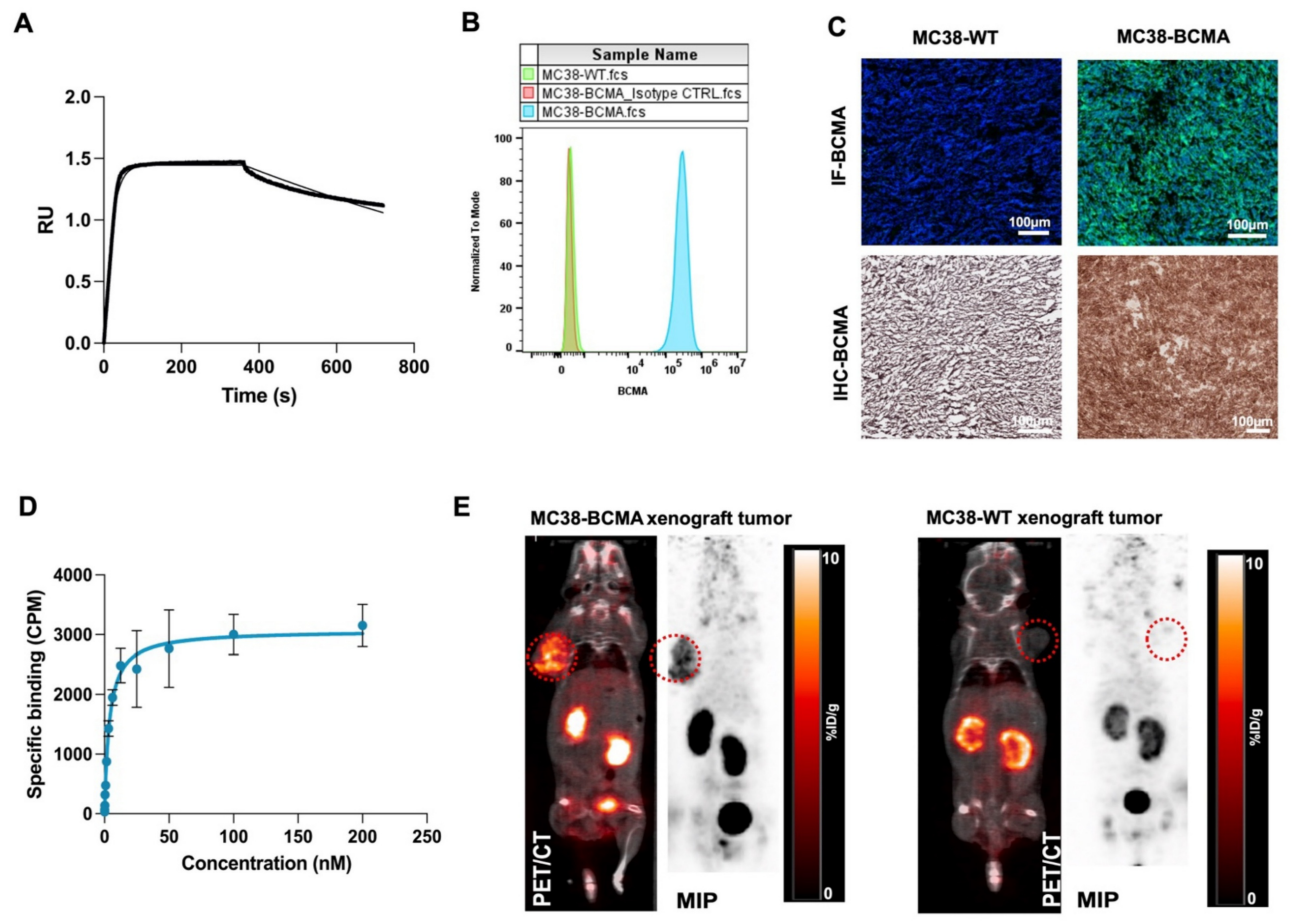
(A) Kinetic binding assay of BCMA nanobody to human BCMA protein measured by biolayer interferometry using 100nM anti-BCMA-nanobody (B) Representative flow cytometry histograms of BCMA expression in MC38-BCMA and MC38-WT cells (C) Immunohistochemistry and Immunofluorescent staining of BCMA in excised tumors (D) Saturation binding assays of [^18^F]FPy-BCMA Nb in MC38-BCMA cells (E) Representative PET/CT coronal images of MC38-BCMA and MC38-WT tumor model (150-250 mm^3^), injected with 3.7 MBq [^18^F]FPy-BCMA-Nb at 2 h post-injection. PET scans are normalized to the same scale. Data are presented as mean ± SD (n = 6 mice per group).

We initially confirmed the affinity of BCMA-Nb for human BCMA using biolayer interferometry with biotinylated human BCMA protein, determining a dissociation constant (K_D_) of 4 nM (Figure 2A). To further assess its cellular binding properties, we conducted studies using MC38 cells (MC38-WT) and MC38 cells transfected with human BCMA (MC38-BCMA). Flow cytometry analysis demonstrated high BCMA expression in MC38-BCMA cells, while MC38-WT cells exhibited no detectable BCMA expression (Figure 2B). These results were further validated in excised tumor sections using immunohistochemistry (IHC) and immunofluorescence (Figure 2C). A saturation binding assay with [^18^F]FPy-BCMA-Nb gave a K_D_ of 3.8 nM, consistent with the interferometry results (Figure 2D), with an estimated of 18,500 BCMA receptors per cell, confirming moderate receptor expression. Furthermore, a competitive cell binding assay demonstrated that [^18^F]FPy-BCMA-Nb binds specifically to MC38-BCMA cells with high affinity (IC_50_ = 5.8 nM), while showing negligible binding to MC38-WT cells (Supplemental Figure S3). *In vitro* uptake studies further verified the tracer's specificity and biological activity, with significantly higher uptake in MC38-BCMA cells compared to MC38-WT cells (Supplemental Figure S4).

To evaluate the pharmacokinetics and tumor-targeting properties of BCMA-Nb *in vivo*, we performed PET imaging in subcutaneous xenograft models (Figure 2E). Imaging results showed significantly higher tumor uptake of [^18^F]FPy-BCMA-Nb in MC38-BCMA xenografts (6 ± 0.8% ID/g) compared to MC38-WT xenografts (1 ± 0.1% ID/g) (P < 0.001, Figure 2E), highlighting the high *in vivo* specificity of the tracer for BCMA-expressing tumors. The use of [^18^F]FPy for nanobody conjugation resulted in a favorable pharmacokinetic profile with rapid clearance from non-target tissues, including the kidneys. Renal uptake decreased to approximately 5-6% ID/g at 2 h post-injection and < 1% ID/g by 3 h. Biodistribution studies confirmed minimal off-target accumulation in vital organs (Figure S5).

### Specificity of [^18^F]FPy-BCMA Nb in Diagnosing Systemic Human MM Models

**Figure 3:**
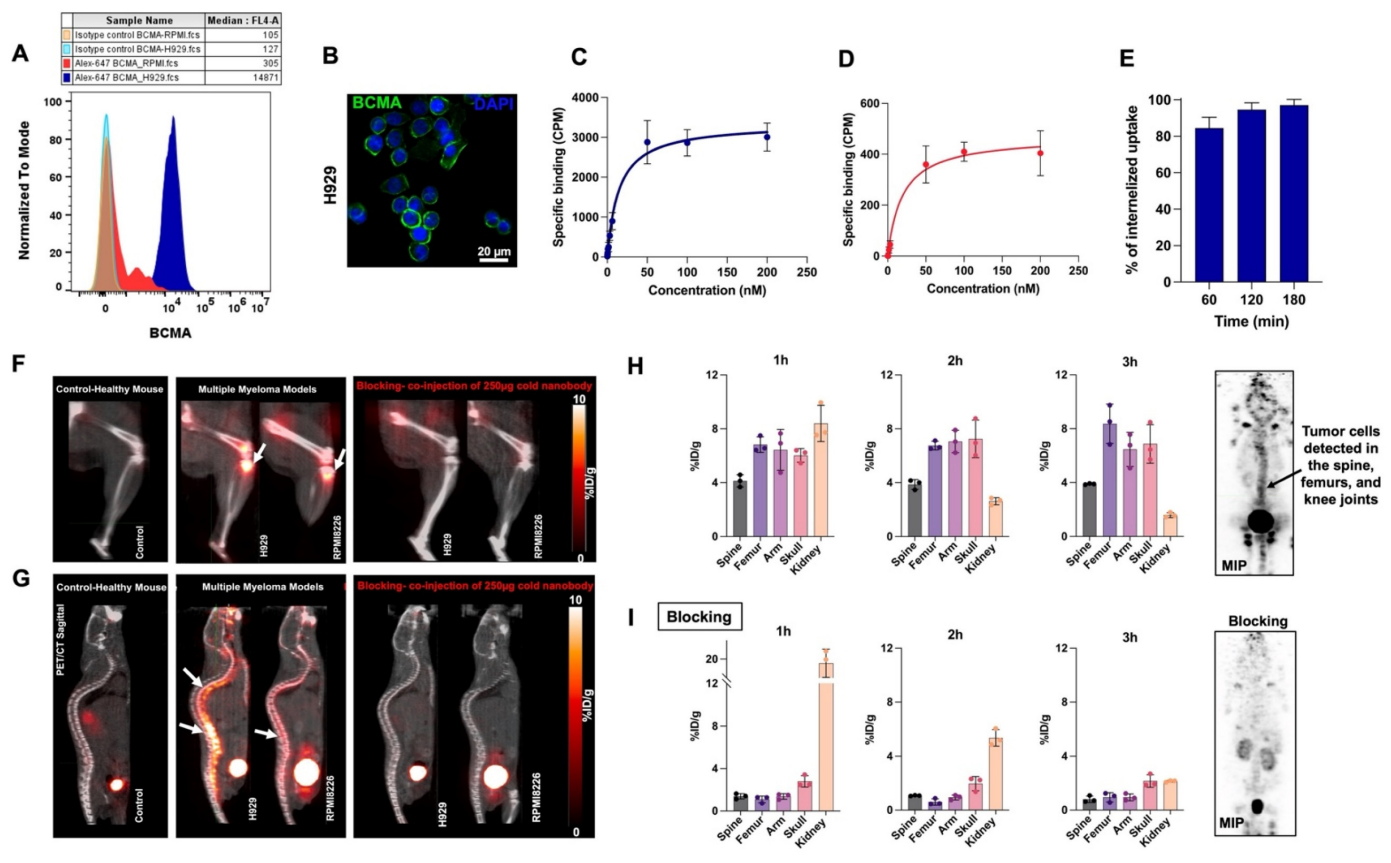
(A) Flow cytometry analysis of BCMA expression in H929 and RPMI8226 cell lines. (B) Immunofluorescent staining of H929 cells using anti-BCMA antibody (green) and DAPI (blue). Saturation binding assay to assess the specific binding and dissociation constant of [^18^F]FPy-BCMA-Nb in (C) H929 and (D) RPMI8226 cells. (E) Internalization of [^18^F]FPy-BCMA-Nb in H929 cells over 180 minutes. (F) 3 h post-injection uptake of [^18^F]FPy-BCMA-Nb in the femur/knee region and (G) spine of the human H929 and RPMI8226 MM model compared to healthy mice and blocking. White arrows indicate tumor cells in the bone marrow. Relative quantitative uptake of the tracer in the H929 human MM model with higher BCMA expression in spine, femur, arm, skull bone marrow, and kidney under (H) pre-blocking and (I) post-blocking conditions**.** Data are presented as mean ± SD (n = 6 mice per group).

Encouraged by the promising results of [^18^F]FPy-BCMA-Nb in imaging subcutaneous BCMA-positive tumors, we switched to a clinically relevant systemic model based on two human MM cell lines, H929 and RPMI8226 administered by tail vein. These cell lines express high and low levels of BCMA, respectively, as validated by flow cytometry and immunofluorescence staining (Figures 3A and 3B). Saturation binding assays (Figures 3C and 3D) showed similar binding affinities (K_D_ of 14.7 nM for H929, 14.3 nM for RPMI8226) but significantly different BCMA receptor densities (20,880 receptors/cell for H929 vs. 5,400 receptors/cell for RPMI8226). Cell uptake and internalization studies demonstrated good tracer retention for up to 3 h (Figure S6) and a consistently high internalization rate across all time points (Figure 3E).

To assess human MM tumor growth in the bone marrow, H929 and RPMI8226 cells were transfected with luciferase, allowing for tumor monitoring via bioluminescence imaging (BLI, Figure S7). The cells were then intravenously injected into NSG mice, where they migrated to the bone marrow and formed multiple metastases within the marrow 18. Once the tumor burden reached sufficient levels on BLI, PET/CT imaging was performed using the [^18^F]FPy-BCMA-Nb up to 3 h post-injection and compared to healthy control mice and a blocking group (Figures 3F and 3G).

Quantitative analysis of the H929 model, which expresses higher levels of BCMA, showed the uptake remained stable at approximately 8 %ID/g in the femur, arm, and skull, and 4% in the spine, showing consistency throughout the 3 h imaging period (Figure 3H). In the RPMI8226 model, with lower BCMA expression, the tracer showed slightly reduced uptake of approximately 4% %ID/g in the femur, arm, and spine, but 8 %ID/g in the skull, where BLI confirmed substantial tumor localization (Figures S7A-B). The tracer demonstrated high tumor specificity, with a tumor-to-muscle ratio of 13.75 and a tumor-to-blood ratio of 57.38, confirming excellent tumor-to-background contrast. Biodistribution at 3 h post-injection showed a non-significant amount of tracer uptake in major organs and a stable uptake of approximately 8% ID/g in bone containing the H929 tumor (Figure S8).

Interestingly, tumor cell migration patterns varied between the two models. In the H929 model, metastasis occurred across multiple bones, forming numerous lesions, while in the RPMI8226 model, metastasis was predominantly localized to the spine and skull, resulting in a large but localized tumor burden.

As free fluoride can accumulate in bone tissue 19, control mice without bone lesions were injected with [^18^F]FPy-BCMA-Nb to ensure that the uptake observed in the models was due to the specificity of the labeled nanobody and not to defluorination. Indeed, bone uptake in the control group was negligible compared to the significant uptake observed in the H929 and RPMI8226 models. To demonstrate specific uptake in bone metastases blocking studies were conducted where [^18^F]FPy-BCMA-Nb was co-injected with 50-fold excess of unlabeled BCMA-Nb. The PET image and quantification showed almost complete blocking of all bone lesions, with levels dropping to less than 1 %ID/g across all lesions, comparable to those observed in healthy controls (Figure 3I and Figure S8B). This further confirms that [^18^F]FPy-BCMA-Nb specifically binds to BCMA-expressing lesions.

### Targeted Radiotherapy with [^131^I]I-BCMA-Nb in a Systemic Human MM Model

**Figure 4:**
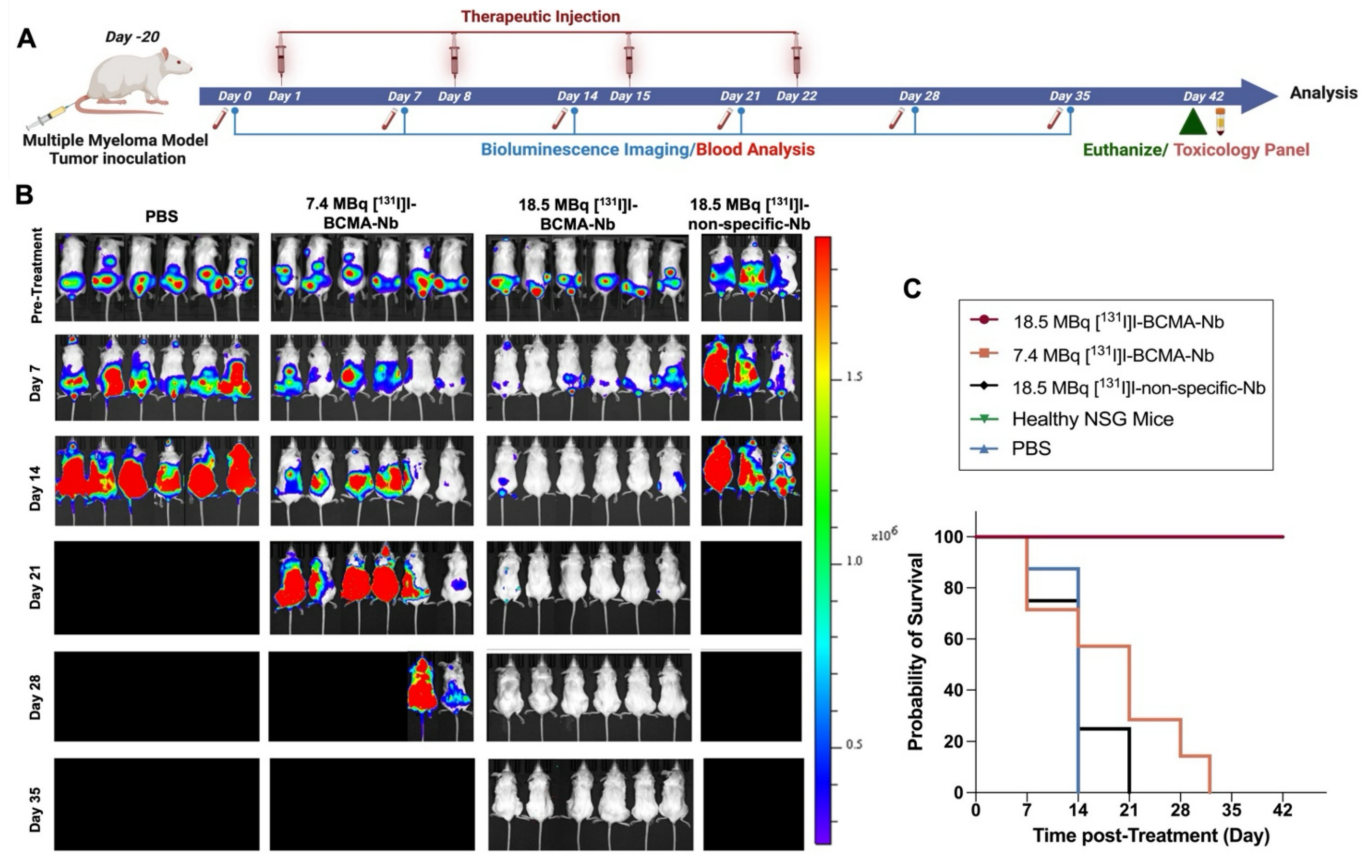
(A) Schematic illustration of the timeline, treatment regimen and efficacy study for the radiotherapy study. (B) Representative BLI images of mice pre- and post-treatment regimen and (C) overall survival percentage across all groups, including Control (PBS), 7.4 MBq and 18.5 MBq of [^131^I]I-BCMA-Nb, compared to 18.5 MBq of [¹³¹I]I-non-specific-Nb treated cohorts, as determined by Kaplan-Meier analysis. Data are presented as mean ± SD (n = 8 mice per group).

The high tumor uptake and rapid internalization of [^18^F]FPy-BCMA-Nb prompted us to assess its therapeutic potential in the H929 highly metastatic, systemic MM model. To confirm that iodination did not interfere with BCMA binding, particularly due to substitution at tyrosine residues that could be critical for binding affinity, we performed a cell binding assay using [^131^I]I-BCMA-Nb. The calculated IC_50_ was 3 nM, comparable to the values obtained when [^18^F]FPy was conjugated to lysine residues, indicating that iodination did not significantly affect binding affinity (Figure S9A). Additionally, a cell uptake and efflux study was conducted to evaluate the extent of radioactivity retention within cells (Figure S9B). This study revealed approximately 50% washout over 6 h incubation, demonstrating reasonable intracellular retention within the cell. To further validate *in vivo* pharmacokinetics of [^131^I]I-BCMA-Nb, we performed single photon emission computed tomography imaging in H929 MM-bearing mice (Figure S10). The spine regions containing MM lesions confirmed by BLI exhibited significantly higher PET signal intensity compared to background tissues. Thyroid uptake was very low, indicating no substantial dehalogenation occurred. Notably, the nanobody displayed rapid renal clearance via the bladder, with no significant kidney retention at 3 h post-injection, supporting its suitability for therapeutic applications. Following these characterization studies, we proceeded to evaluate the therapeutic efficacy of [^131^I]I-BCMA-Nb in four treatment groups: (1) a control group receiving PBS injections, (2) a group treated with 7.4 MBq of [^131^I]I-BCMA-Nb, (3) a group receiving 18.5 MBq of [^131^I]I-BCMA-Nb, and (4) a group administered 18.5 MBq of [^131^I]I-non-specific-Nb, which lacks BCMA binding. Given the rapid clearance of nanobodies and based on previous studies on other [^131^I]I-labeled nanobodies 15, 20, we designed a treatment regimen consisting of four weekly injections over four weeks (Figure 4A). Therapeutic response and tumor progression were monitored weekly through BLI and [^18^F]FDG-PET imaging. As expected, mice in the control group exhibited aggressive tumor progression, reaching humane endpoints necessitating euthanasia by day 14 post-treatment initiation (Figure 4B). Similarly, the group treated with 18.5 MBq of [^131^I]I-non-specific-Nb showed no therapeutic effect, confirming the necessity of BCMA targeting. In contrast, the group receiving 7.4 MBq of [^131^I]I-BCMA-Nb displayed partial tumor regression and prolonged survival compared to the control and non-specific groups (Figure 4C). However, the 18.5 MBq [^131^I]I-BCMA-Nb group demonstrated significant tumor reduction, particularly after the third and fourth treatment sessions (Figure 4B). While in the 7.4 MBq group by the third session, 60% of mice were disease free by BLI, in the 18.5 MBq group 98% of lesions had disappeared, indicating near-complete disease control.

The fourth treatment session had minimal impact on the 7.4 MBq group, which ultimately reached end point, while the 18.5 MBq group achieved 100% remission (Figure 4C). Importantly, follow-up assessments one week after the final fourth treatment revealed no evidence of recurrence or tumor regrowth in this cohort, demonstrating the treatment's ability to effectively eliminate BCMA-positive MM cells and prevent relapse (Figure 4B). Kaplan-Meier survival analysis further confirmed a dose-dependent survival benefit following [^131^I]I-BCMA-Nb therapy. Mice treated with 18.5 MBq [^131^I]I-BCMA-Nb exhibited a significant prolongation of survival compared with the PBS control group (log-rank χ² = 12.22, df = 1, p = 0.0005; Gehan-Breslow-Wilcoxon χ² = 11.83, p = 0.0006). The median survival for PBS-treated mice was 14 days, whereas the median survival for the 18.5 MBq group was not reached, indicating a sustained therapeutic effect. The corresponding hazard ratio for death was 0.037 (95% CI 0.0058-0.234), reflecting a markedly reduced mortality risk. In contrast, mice receiving 7.4 MBq [^131^I]I-BCMA-Nb demonstrated a modest but non-significant improvement in survival (median 21 vs. 14 days for PBS; log-rank χ² = 2.23, p = 0.135). Together, these findings indicate that therapeutic efficacy and survival benefit are dose dependent, with the higher-dose [^131^I]I-BCMA-Nb achieving statistically significant and durable disease control.

**Figure 5:**
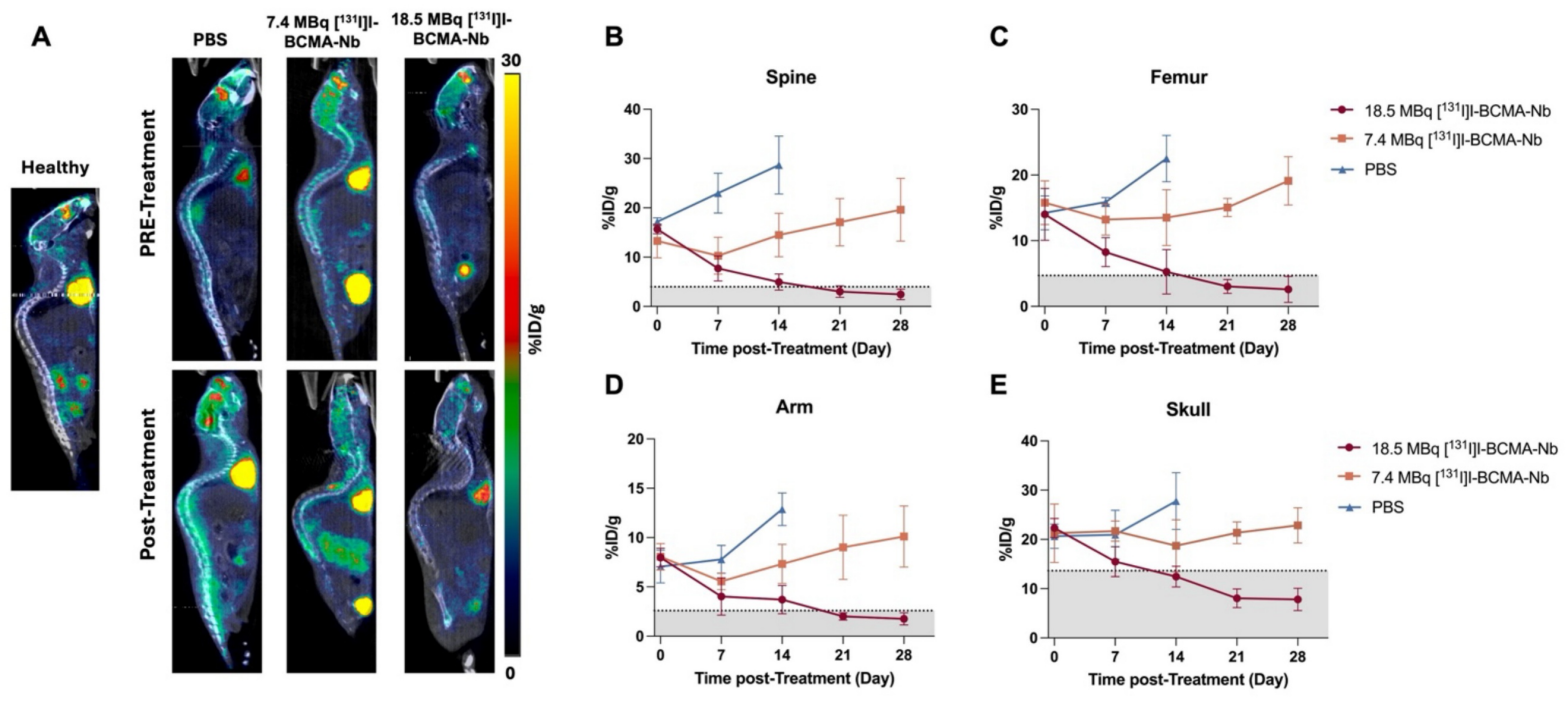
(A) Representative Sagittal [^18^F]FDG PET images of healthy mice compared to treatment groups (PBS, 7.4 MBq and 18.5 MBq of [^131^I]I-BCMA-Nb,) acquired pre- and post-treatment regimen. Quantitative analysis of [^18^F]FDG uptake in bone marrow of the (B) spine, (C)femur, (D) arm, and (E) skull across all groups pre-, during and post-treatment. Gray areas are defined as baseline based on [^18^F]FDG of healthy mice. Data are presented as mean ± SD (n = 8 mice per group).

To further assess therapeutic response, we used [^18^F]FDG PET and imaging, the tracer used clinically for evaluating tumor metabolism. Baseline scans of healthy mice revealed expected [^18^F]FDG distribution, including low skeletal uptake and high activity in the brain and heart (Figure 5A). This reference enabled differentiation between normal tracer uptake and MM-related metabolic activity. In the PBS-treated MM-bearing group, [^18^F]FDG uptake increased markedly over time in the spine, femur, skull, and forelimbs, reflecting aggressive tumor expansion within the bone marrow (Figure 5A). Mice treated with 7.4 MBq [^131^I]I-BCMA-Nb exhibited a slower increase in [^18^F]FDG uptake, particularly in skull lesions, but spinal involvement persisted. In contrast, in the 18.5 MBq treatment group e [^18^F]FDG uptake was comparable to healthy controls, indicating near-complete metabolic suppression of MM lesions. Quantitative analysis of [^18^F]FDG uptake (Figure 5B) further supported these observations, emphasizing the dose-dependent efficacy of [^131^I]I-BCMA-Nb in eliminating BCMA-positive malignant cells and controlling their tumor metabolism.

To assess post-treatment BCMA expression, we performed [^18^F]FPy-BCMA-Nb PET imaging at the end of the study in the main treatment group. As shown in Figure S11, mice receiving 18.5 MBq [^131^I]I-BCMA-Nb showed no detectable tracer uptake in the lumbar spine, skull, femur, or other skeletal sites, indicating the absence of BCMA-positive lesions and consistent with a complete therapeutic response.

**Figure 6:**
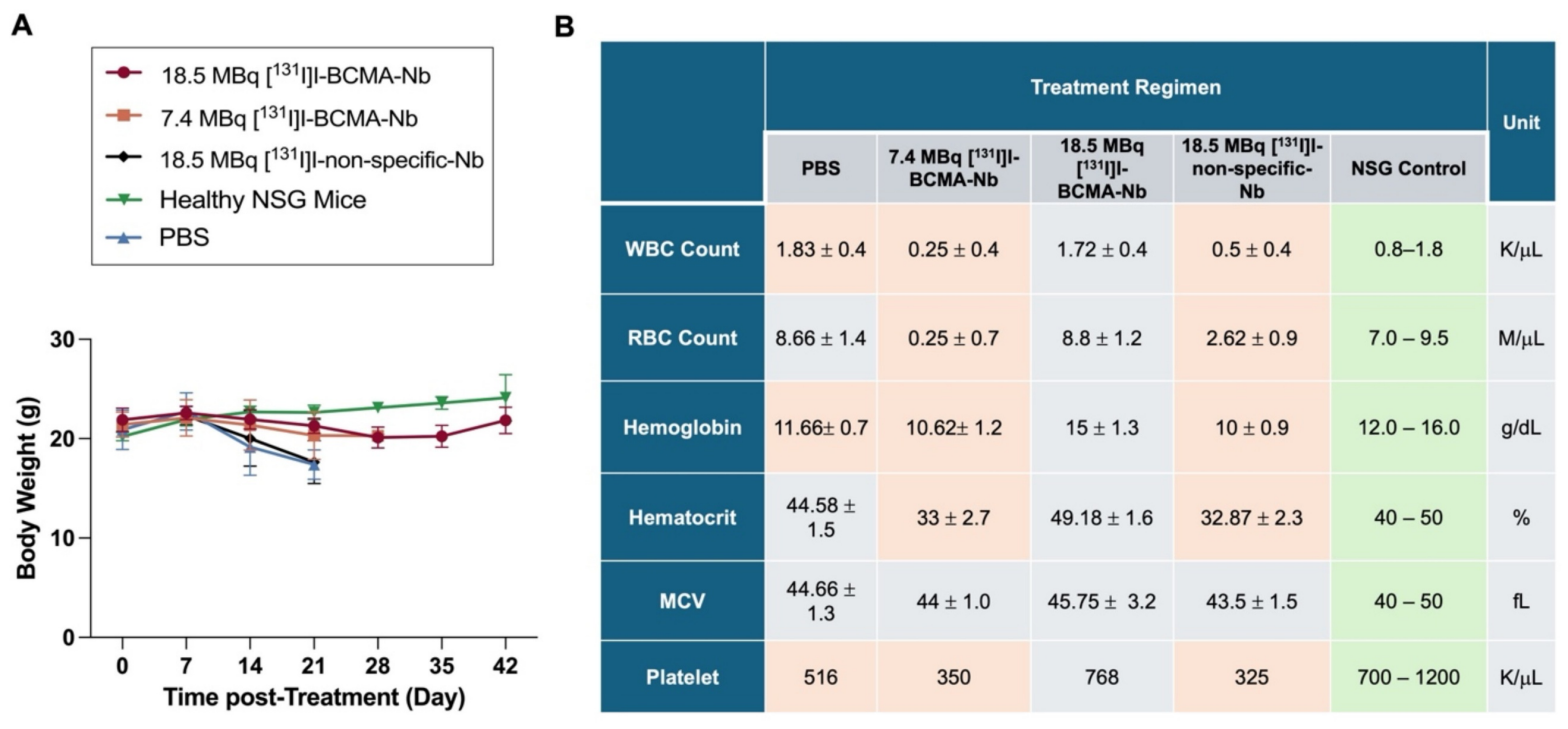
Changes in (A) body weight during the treatment regimen. (B) Complete cell blood counts for mice pre- and post-treatment in all groups including PBS, injected with 7.4 MBq and 18.5 MBq of [^131^I]I-BCMA-Nb and 18.5 MBq of [^131^I]I-non-specific-Nb. Orange-shaded cells indicate values significantly different from standard NSG mouse ranges (p<0.05). Data are presented as mean ± SD (n = 8 mice per group).

To evaluate potential systemic toxicity, we monitored body weight changes, blood analysis and biochemistry panels throughout the study (Figure 6). Mice in the PBS and 18.5 MBq [^131^I]I-non-specific-Nb groups exhibited significant and rapid weight loss during the first two weeks, culminating in euthanasia due to disease progression. In contrast, mice treated with 7.4 or 18.5 MBq of [^131^I]I-BCMA-Nb exhibited only minimal weight loss, which was not statistically significant compared to healthy controls (Figure 6A). These findings suggest that tumor regression contributed to weight stabilization and recovery, and that the treatment regimen did not induce substantial systemic toxicity. Blood analyses are summarized in Figure 6B and Table S1. In the control, 7.4 MBq [^131^I]I-BCMA Nb, and 18.5 MBq [^131^I]I-non-specific-Nb groups, significant decreases in white blood cells, red blood cells, hemoglobin, hematocrit, and platelets were noted, reflecting the progression of multiple myeloma in the bone marrow. In contrast, no significant differences were observed in these hematologic parameters for the 18.5 MBq [^131^I]I-BCMA-Nb group, suggesting that effective tumor reduction enabled bone marrow recovery and restoration of normal hematopoietic function.

**Figure 7:**
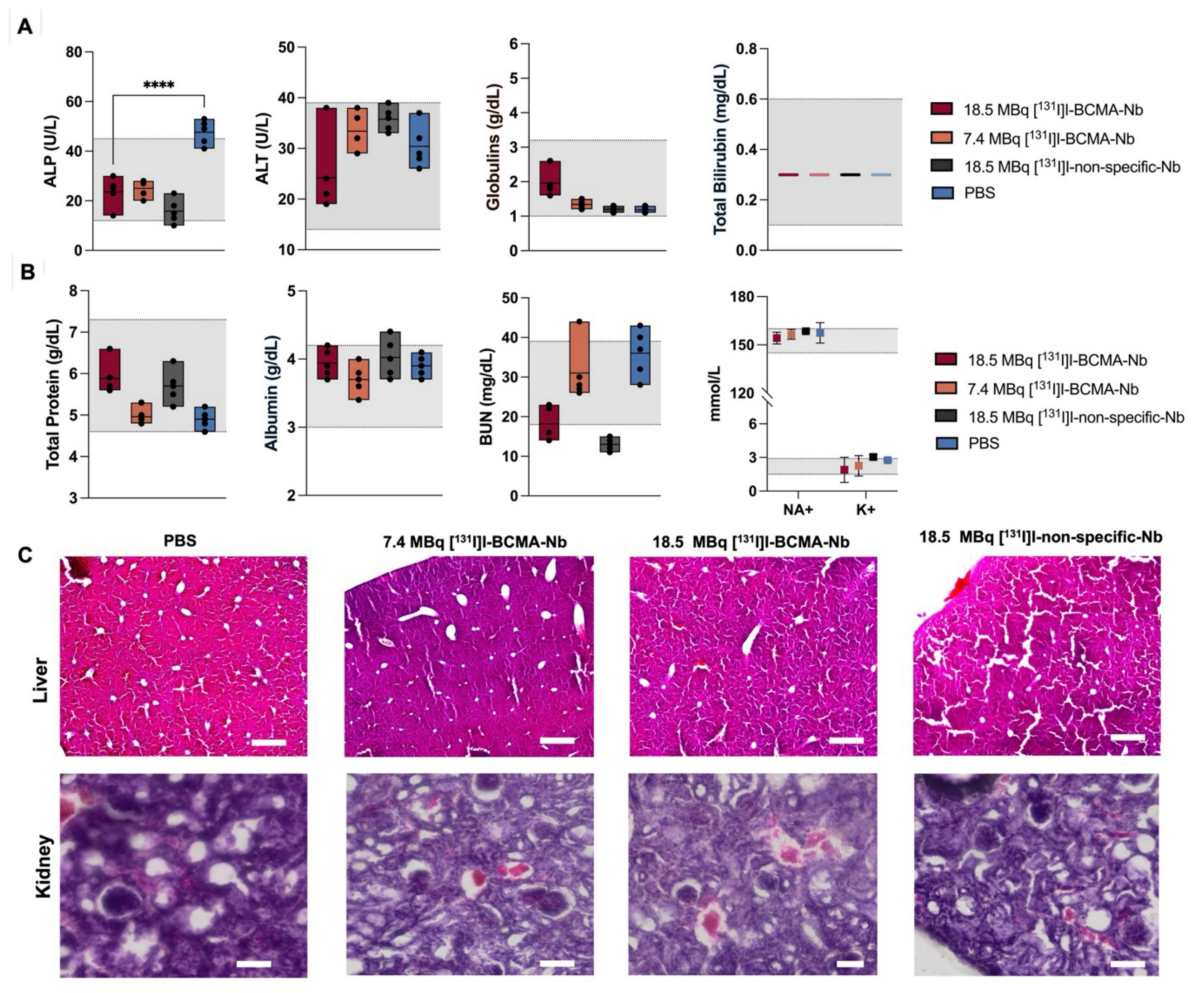
Biochemical analyses of (A) liver and (B) kidney function, as well as (C) H&E staining of liver and kidney in all groups post-treatment. Gray area represents the reference ranges for healthy NSG mice. Data are presented as mean ± SD (n = 5 mice per group).

Biochemical analyses, including standard liver function tests (ALP, ALT, globulin, and total bilirubin), revealed no significant changes attributable to the therapy (Figure 7A). All values for the treated groups fell within the established reference ranges for healthy NSG mice (denoted by the shaded area). In contrast, the control cohort exhibited a marked increase in ALP, likely reflecting the severe tumor burden in the bone marrow and its secondary impact on overall health and liver function. Additionally, the assessment of serum biochemical markers associated with kidney function and overall metabolic status remained within normal reference ranges (Figure 7B and Figure S12). Moreover, histopathological examination (H&E staining) of liver and kidney tissues across all cohorts (Figure 7C) confirmed the absence of significant treatment-associated toxicity in these organs.

**Figure 8:**
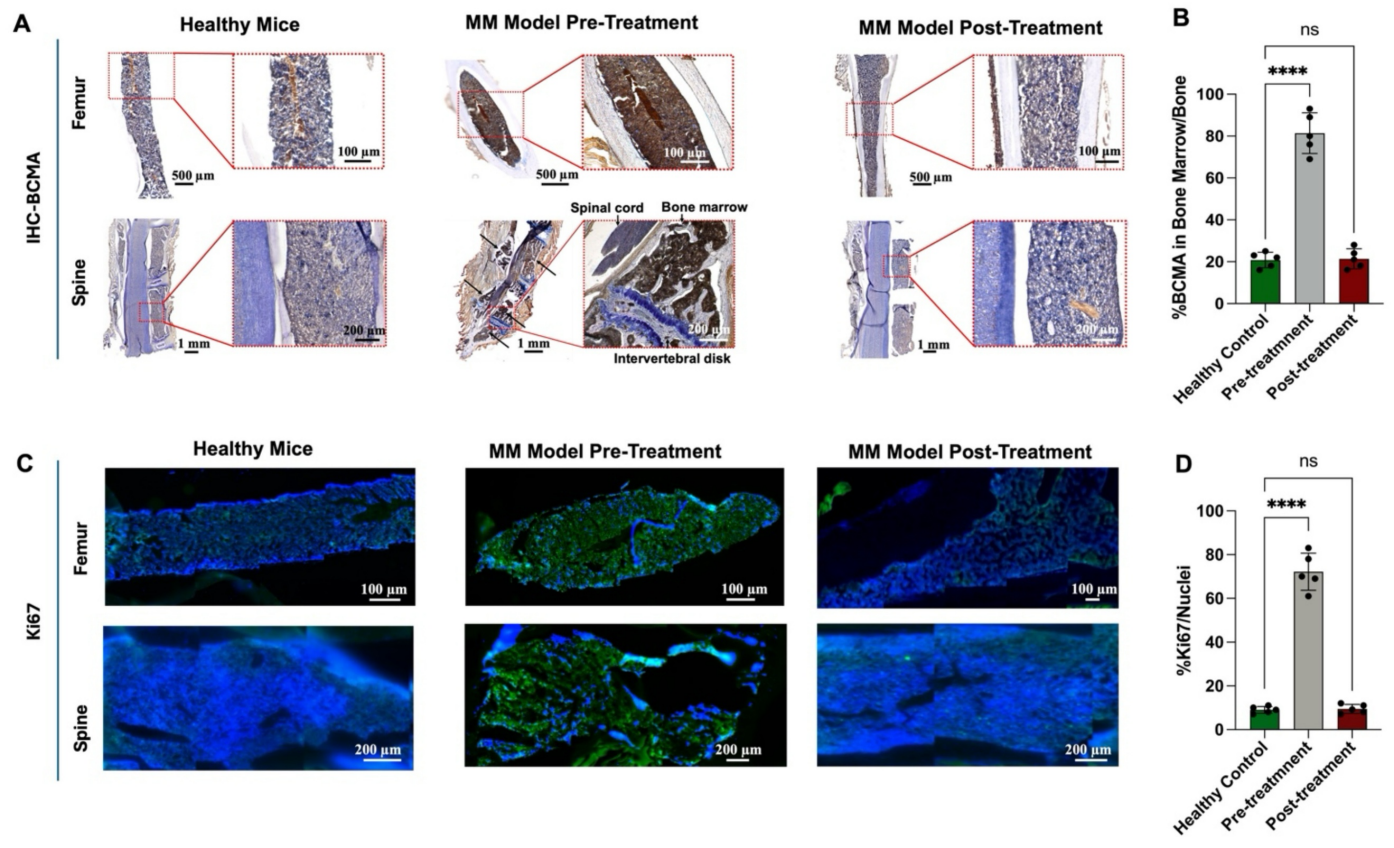
(A) Immunohistochemistry staining and (B) quantitative analysis of BCMA, (C) immunofluorescence staining for and (D) quantitative analysis of Ki67 proliferation marker in femur and spine bone marrow sections of H929 human MM-bearing mice, before and after treatment, compared with healthy mice.

We also performed IHC staining to evaluate BCMA expression in bone marrow samples before and after treatment (Figure 8A). Before treatment, BCMA expression was significantly elevated, showing an 80% increase relative to healthy bone tissue. However, following treatment with 18.5 MBq [^131^I]I-BCMA-Nb, BCMA levels dropped to 20%, closely resembling those observed in healthy mice (Figures 8A-B). Further analysis of Ki-67, a marker of cell proliferation, demonstrated that mice treated with 18.5 MBq [^131^I]I-BCMA-Nb exhibited significantly lower Ki-67 expression compared to pre-treatment levels, with values comparable to those of healthy controls (Figure 8C). Quantification of Ki-67-positive cells relative to total nuclei revealed a 70% increase in proliferation before treatment, consistent with active MM cell division. Post-treatment, proliferation levels declined to approximately 10%, aligning with the physiological levels observed in healthy bone marrow (Figure 8D). These findings confirm a significant reduction in BCMA-expressing MM cells after treatment with [^131^I]I-BCMA-Nb, highlighting its selectivity for MM, enabling bone marrow recovery.

We also looked at CD45 immunofluorescence staining of femur and vertebral bone marrow, which showed dense CD45⁺ hematopoietic infiltrates in control mice but a near-complete absence of CD45⁺ cells in the 18.5 MBq [^131^I]I-BCMA-Nb-treated group, consistent with effective depletion of BCMA-expressing myeloma cells from the marrow (Supplemental Fig. S13).

## Discussion

BCMA has emerged as one of the most clinically transformative targets in multiple myeloma (MM), underpinning the success of CAR-T cells, bispecific antibodies, and antibody-drug conjugates 21, 22. However, these therapies highlight a growing need for tools that can noninvasively assess BCMA expression, quantify disease burden, and guide therapeutic decision making. Imaging BCMA provides a means to overcome the sampling limitations of bone marrow biopsies, capture spatial heterogeneity, and identify sites of active disease throughout the skeleton. Likewise, selective radiotherapeutic delivery to BCMA-expressing plasma cells offers a complementary strategy for patients who relapse after immunotherapies or harbor refractory disease. Developing radiopharmaceuticals that both visualize and eradicate BCMA-positive lesions therefore represents a major opportunity to refine MM diagnosis, staging, and treatment.

Several research groups have previously explored BCMA-targeted PET tracers for MM imaging. Wei et al. developed [^68^Ga]Ga-labeled BCMA nanobodies that enabled visualization of BCMA expression in preclinical MM models, while Thomas et al. introduced a [^64^Cu]Cu-labeled anti-BCMA nanoparticle-antibody construct that improved lesion detection in the spine and femur of MM-bearing mice 13, 23. More recently, Song et al. reported a [^68^Ga]Ga -labeled BCMA-binding peptide [^68^Ga]Ga-DOTA-BP1) with specific uptake in BCMA-positive xenografts, and Wang et al. extended the field by developing an [^89^Zr]Zr-labeled full-length anti-BCMA monoclonal antibody for immunoPET, demonstrating selective targeting in MM animal models and first-in-nonhuman-primate imaging 24, 25. While these studies emphasize the growing interest in BCMA-targeted imaging, they also highlight recurring limitations associated with radiometal-based tracers. Most reported approaches rely on radiometal-based labeling, which imposes intrinsic pharmacokinetic constraints including slow blood clearance, modest tumor-to-background ratios, and substantial renal retention driven by proximal tubular reabsorption of charged metal-chelate complexes. These characteristics limit the sensitivity of such tracers for detecting small or early marrow lesions and restrict their suitability for therapeutic pairing due to nephrotoxicity concerns.

Our halogen-based strategy represents a mechanistic advancement: [^18^F]FPy and [^131^I]I labeling produced nanobody constructs with rapid systemic clearance, dramatically reduced kidney uptake, and significantly improved image contrast. The nanobody format further supports fast tumor penetration and enables same-day imaging, providing workflow advantages over full antibodies or nanoparticle systems. In addition, the mild, two-step room-temperature [^18^F]fluorination chemistry and broad availability of cyclotron-produced [^18^F]fluoride enhance the practicality and scalability of clinical translation compared with radiometal-chelation approaches. Most importantly, the therapeutic efficacy demonstrated by the [^131^I]-labeled nanobody analog, achieving complete remission in an aggressive systemic MM model, establishes a true theranostic platform that integrates high-performance BCMA imaging with effective targeted radionuclide therapy, a capability not demonstrated with prior BCMA tracers. These distinctions support the novelty and translational potential of this halogen-labeled BCMA nanobody.

To better understand the molecular determinants of tracer performance, we also examined binding characteristics across different model systems. An interesting experimental observation in the saturation assays was that the apparent K_D_ values of [^18^F]FPy-BCMA-Nb were higher in H929 and RPMI-8226 cells compared with measurements obtained by biolayer interferometry using recombinant BCMA protein or MC38-BCMA-transfected cells. This difference may reflect post-translational modifications of BCMA (such as glycosylation or phosphorylation) that could alter epitope conformation and affect the binding interactions. Additionally, BCMA expressed on MM cell lines like H929 and RPMI8226 exist within a complex cell surface environment, interacting with various ligands such as APRIL and BAFF. These interactions may induce conformational shifts or partially mask certain epitopes, leading to a higher K_D_ value when compared to engineered models like MC38-BCMA cells, where BCMA expression occurs in a more controlled environment 26, 27. The nanobody also displayed a high internalization rate, an advantageous feature for therapeutic applications because intracellularly retained isotopes achieve greater radiotoxic effect 28, 29. Although [^18^F]FPy-BCMA-Nb PET is not specifically designed for MRD detection, the imaging sensitivity demonstrated in this study is notable. The tracer successfully visualized early lesions in the RPMI-8226 systemic MM model, despite these cells expressing only ~5,400 BCMA receptors per cell and being introduced at a very low burden (1 × 10⁶ cells administered intravenously). Detecting lesions derived from such low receptor copy numbers and small initial tumor inocula highlights the robustness of nanobody-based targeting and emphasizes the potential of [^18^F]FPy-BCMA-Nb for identifying low-volume or early-stage disease, even if it is not optimized for formal MRD assessment. We also evaluated factors that might influence biodistribution or target engagement. Under blocking conditions, kidney uptake of [¹⁸F]FPy-BCMA-Nb increased slightly, consistent with nanobody physiology: reduced receptor engagement leaves more unbound tracer available for renal filtration. Soluble BCMA (sBCMA) could, in principle, compete with targeting, but the injected nanobody dose (~10-15 µg/mouse) corresponds to nanomolar plasma concentrations likely in molar excess over circulating sBCMA in this model. PET imaging showed no evidence of antigen-sink effects. Nonetheless, sBCMA may affect tracer behavior in patients with very high circulating levels, and this will be evaluated in future translational studies with our clinical candidate.

The preserved affinity of [^131^I]I-BCMA-Nb (IC₅₀ ≈ 3 nM) together with strong lesion uptake on SPECT and negligible thyroid signal indicates that direct iodination did not compromise target engagement or *in vivo* stability. Similar behavior has been reported for HER2-directed sdAbs that retain high-affinity binding after iodination with selective tumor uptake 15, 20. Therapeutically, fractionated weekly administration of [^131^I]I-BCMA-Nb produced clear activity-dependent responses, culminating in near-complete disease control at the higher activity. Fractionation is consistent with long-standing radioimmunotherapy experience, where dividing the administered activity across multiple treatments can enhance tumor dosing while mitigating marrow exposure, an approach supported both by clinical [^131^I]I-antibody studies and pharmacokinetic modeling of ^131^I-labeled immunoconjugates 30-32.

Although [^18^F]FDG PET remains the clinical standard for assessing metabolic activity in MM, its sensitivity and specificity are imperfect, as FDG uptake can be confounded by post-treatment inflammation, heterogeneous metabolic activity across skeletal sites, and physiological bone marrow uptake, factors that contribute to both false positives and false negatives 33. In contrast, [^18^F]FPy-BCMA-Nb PET directly visualizes BCMA-expressing myeloma cells, enabling more accurate delineation of true disease burden and therapeutic response. The performance of [^18^F]FPy-BCMA-Nb in systemic MM models reinforces its utility, demonstrating precise mapping of heterogeneous skeletal involvement that often eludes standard imaging. Given the well-documented discordance between focal lesions and routine biopsy sites, such BCMA-specific imaging may reduce false-negative biopsies, improve assessment of minimal residual disease, and better inform eligibility for BCMA-directed therapies, ultimately supporting more accurate and individualized clinical decision-making.

It is important to note that although both [^131^I]I-BCMA-Nb and [^18^F]FPy-BCMA-Nb use non-residualizing halogen labels, small differences in their *in vivo* kinetics are still expected due to differences in radiolabel chemistry. Factors such as dehalogenation susceptibility, lipophilicity of the prosthetic group, intracellular catabolism, and thyroid trapping of free iodine can influence clearance patterns and apparent retention. These considerations have been reported for other nanobody and antibody-fragment tracers and should be taken into account when comparing quantitative imaging data to therapeutic behavior.

A limitation of this study is the use of murine models is the use of a murine models, which do not fully replicate the complexity of the human bone marrow microenvironment or immune contexture. The cytokine milieu, stromal architecture, marrow vascularization, and composition of resident and infiltrating immune cells differ substantially between mice and humans. These factors influence plasma cell survival, BCMA expression dynamics, immune-mediated clearance mechanisms, and marrow sensitivity to radiation. As such, the therapeutic and imaging performance observed here may not directly predict behavior in the more heterogeneous and immunologically complex human marrow niche. Ongoing work with our next-generation nanobody candidate will therefore incorporate additional preclinical platforms, including humanized and orthotopic models, to better capture human-relevant biology prior to translation.

Taken together, this study establishes a proof-of-concept halogen-based nanobody platform for precise BCMA imaging and targeted radionuclide therapy in multiple myeloma, providing a foundation for the development of future clinical candidates. The combination of rapid clearance, low renal retention, and high lesion specificity distinguishes this tracer from prior BCMA agents and enables sensitive detection of low-burden or early-stage disease. The therapeutic efficacy of the [^131^I]I-labeled analog further supports the feasibility of a true nanobody-based theranostic strategy for MM. Beyond oncology, the ability to selectively target long-lived BCMA-expressing plasma cells may broaden the utility of this approach to autoimmune disorders driven by refractory autoreactive plasma cells. Together, these findings highlight the translational potential of BCMA-directed nanobody radiopharmaceuticals and lay the foundation for future clinical development.

## Supplementary Material

Supplementary figures and table.
