# Supplementary material for: Precision radiolabeled B-cell maturation nanobody for targeted PET imaging and radioligand therapy of disseminated multiple myeloma
Source: Theranostics. 2026 Jan 1;16(6):2748–63. doi: 10.7150/thno.126920 (PMC12775650; doi:10.7150/thno.126920)
Supplement: Supplementary file 1 — Supplementary figures and table. [file thnov16p2748s1.pdf]

Supplementary Information File

Precision Radiolabeled B-Cell Maturation Nanobody for Targeted PET Imaging and Radioligand Therapy of Disseminated Multiple Myeloma

Behnaz Ghaemi[1], Colleen P. Olkowski[1], Falguni Basuli[2], Jianfeng Shi[2], Ryan Young[3], Dickran Kazandjian[4], Ola Landgren[4], Elizabeth Hill[3], Peter L. Choyke[1] and Orit Jacobson[1*]

[1]Molecular Imaging Branch, Center for Cancer Research, National Cancer Institute, NIH, Bethesda, Maryland, 20892; [2]Chemistry and Synthesis Center, National Heart, Lung, and Blood Institute, NIH, Rockville, Maryland, 20850; [3]Lymphoid Malignancies Branch, Center for Cancer Research, National Cancer Institute, NIH, Bethesda, Maryland, 20892; [4]Sylvester Myeloma Institute, Sylvester Comprehensive Cancer Center, Department of Medicine, Myeloma Division University of Miami, Florida, 33146

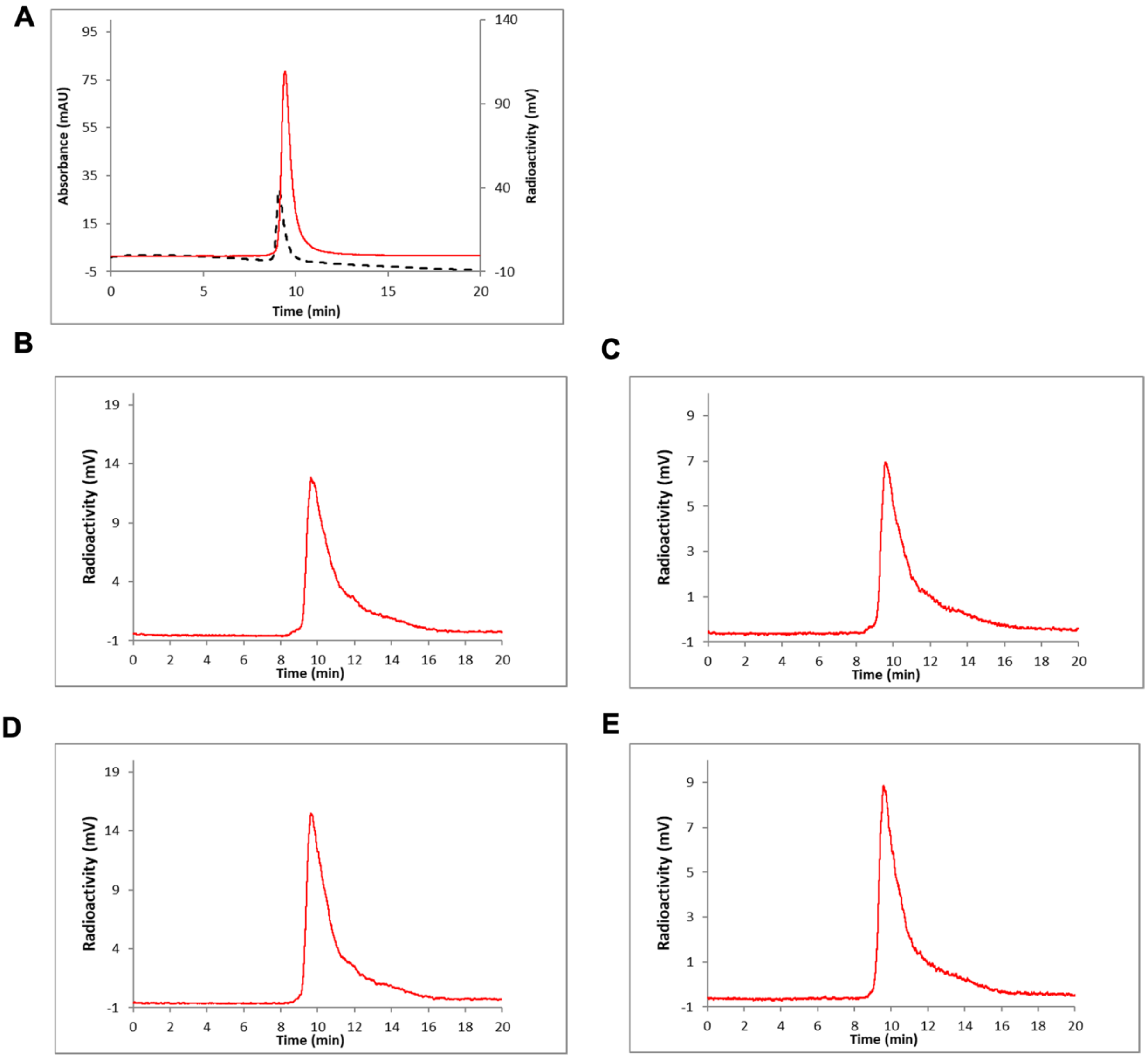

Supplemental Figure S1. HPLC chromatogram of [^{18}F]F-FPy-BCMA-Nb in (A) PBS, mouse serum (B) 0 hour, (C) 3 hour; Whole human serum (D) 0 hour and (E) 3 hours.

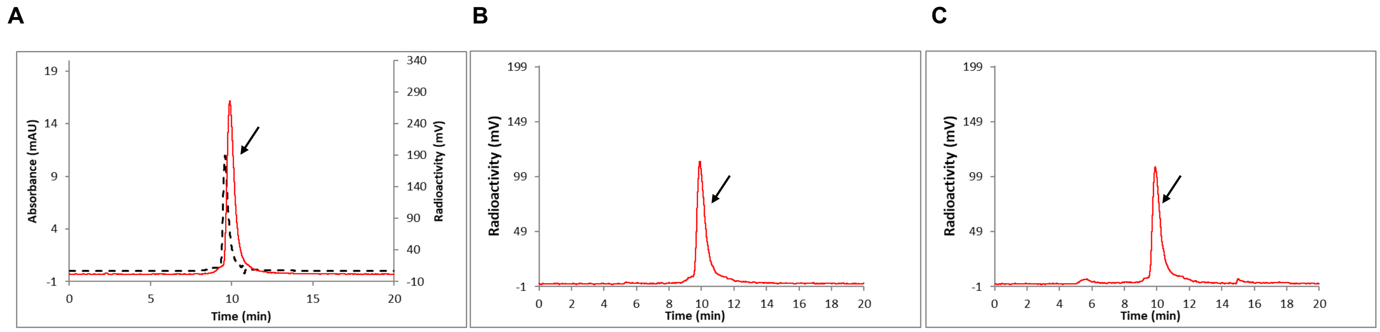

Supplemental Figure S2. (A) HPLC chromatogram of [^{131}I]I-BCMA-Nb in PBS and mouse serum (B) 0 hour and (C) 3 hours.

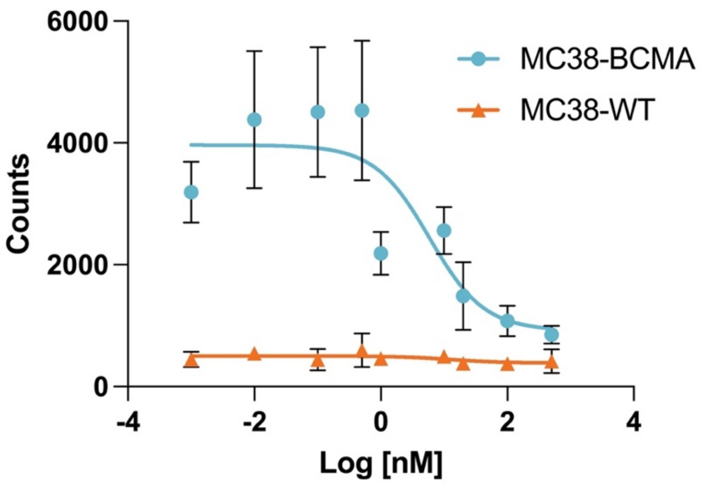

Supplemental Figure S3. Competitive cell-binding assays, [^{18}F]F-FPY-BCMA-Nb as competitor for 1 hour incubation time in MC38-WT and MC38-BCMA cells.

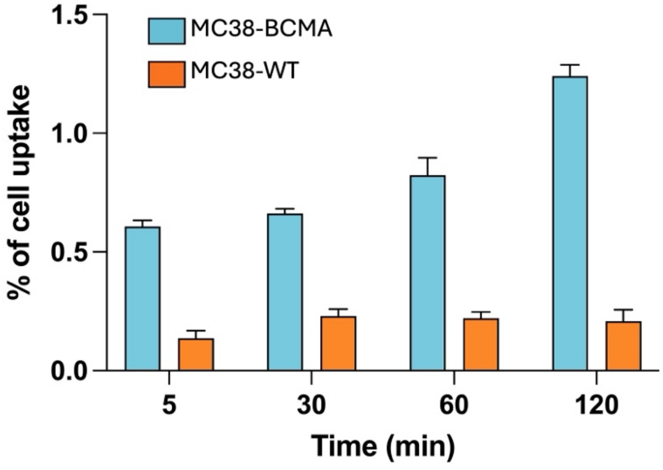

Supplemental Figure S4. Cell uptake of [^{18}F]F-FPY-BCMA-Nb up to 2 hours post incubation with MC38-WT and MC38-BCMA cells.

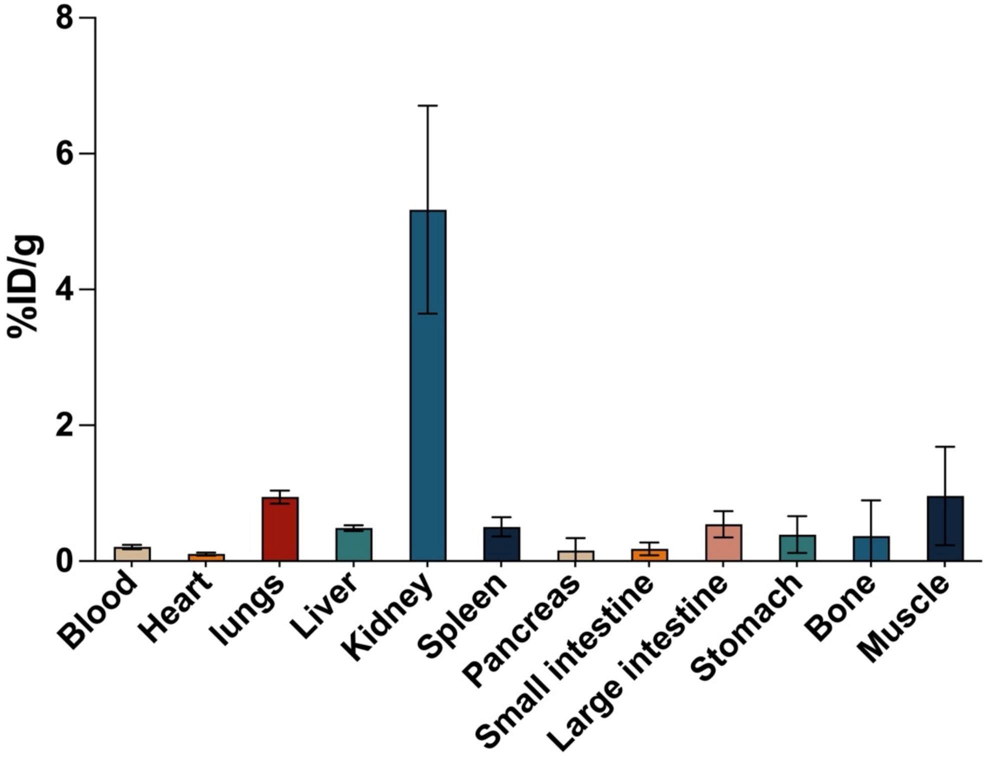

Supplemental Figure S5. Biodistribution analysis of [^{18}F]F-FPY-BCMA-Nb at 2 hours post-injection in athymic nude mice.

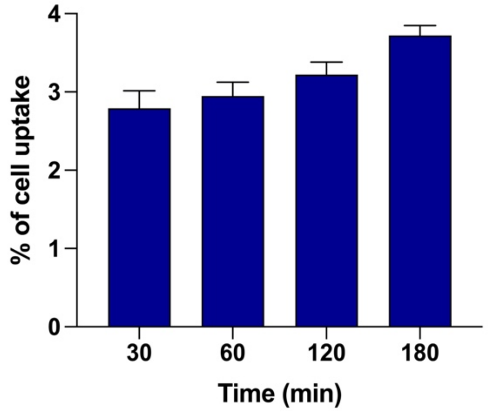

Supplemental Figure S6. Cell uptake of [^{18}F]F-FPY-BCMA-Nb up to 3 hours post incubation with H929 cells.

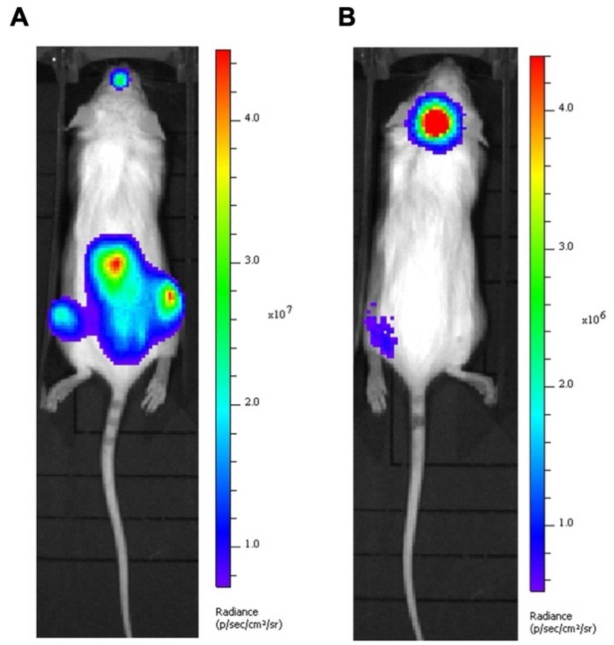

Supplemental Figure S7. Representative bioluminescence images of mouse with (A) H929 and (B) RPMI8226 tumors.

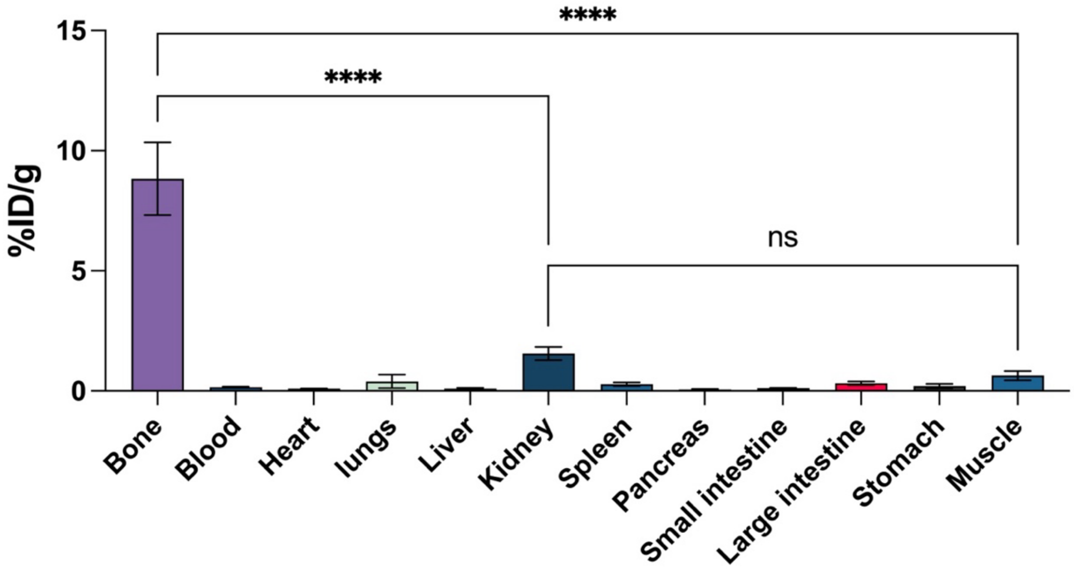

Supplemental Figure S8. Biodistribution analysis of [^{18}F]F-FPY-BCMA-Nb at 3 hours post-injection in systemic MM-bearing athymic nude mice.

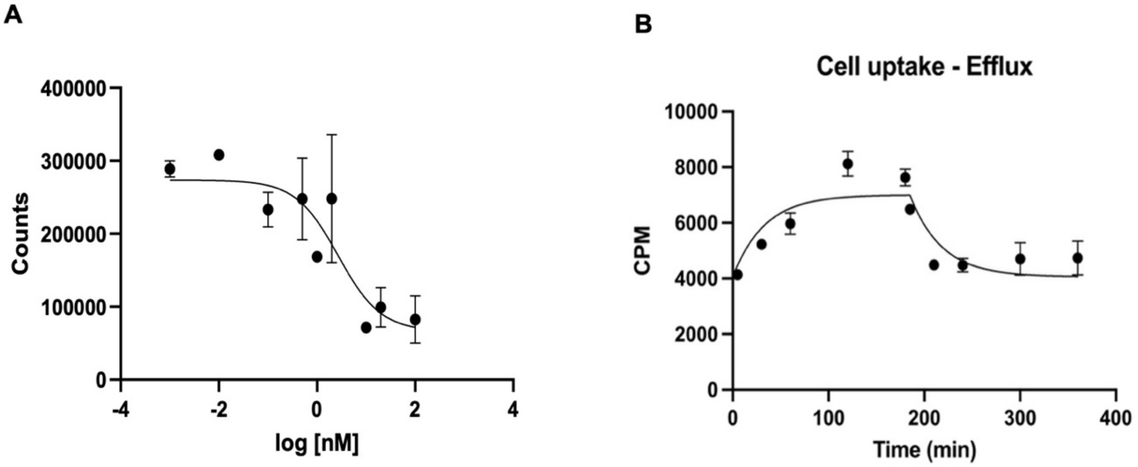

Supplemental Figure S9. (A) Competitive cell-binding assay and (B) Cell uptake and efflux studies of [^{131}I]I-BCMA-Nb in H929 cells.

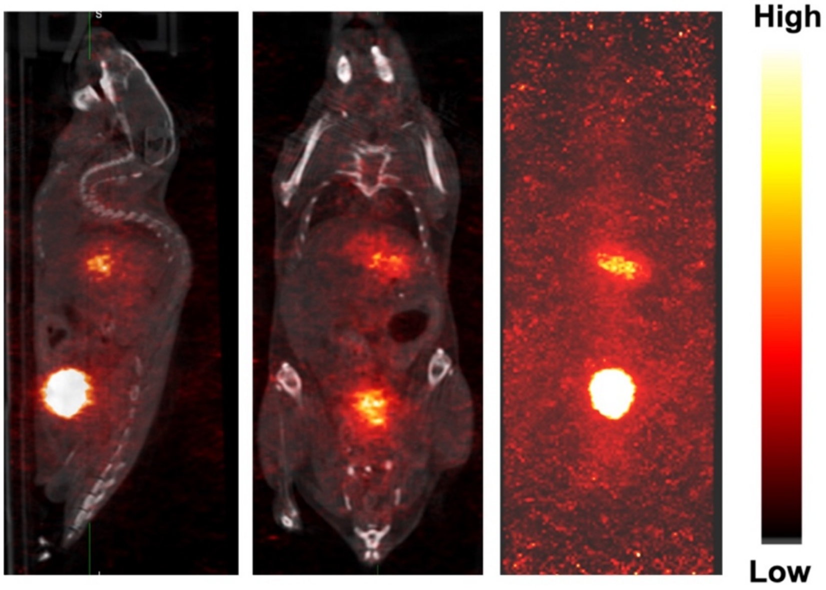

Supplemental Figure S10. Representative SPECT/CT images of a mouse with sagittal view (left), coronal view (middle) and SPECT MIP (right) of systemic H929 MM model, injected with 18.5 MBq [^{131}I]I-BCMA-Nb, highlighting radiotracer uptake in the spine, with no detectable thyroid uptake, at 3 hours post-injection.

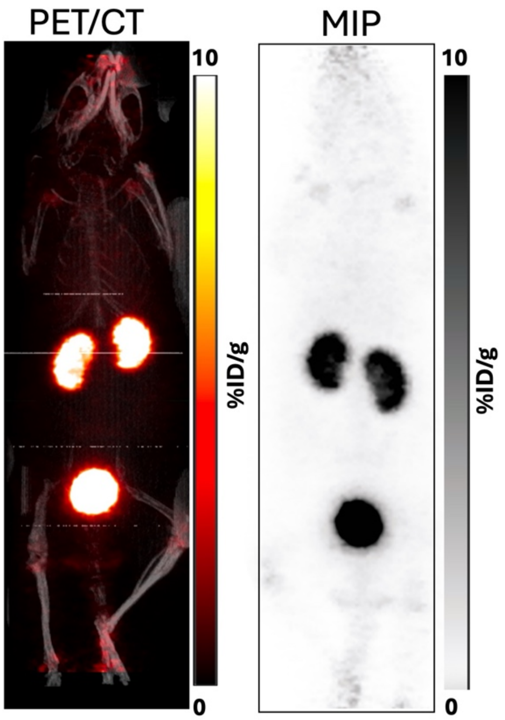

Supplemental Figure S11. Representative PET/CT and MIP images in MM-bearing mice after treatment with 18.5 MBq [^{131}I]I-BCMA-Nb, acquired using [^{18}F]FPy-BCMA-Nb PET imaging to evaluate the presence of any residual or newly developed lesions following therapy. The scans were taken on Mediso nanoPET/CT system at 2 hours post-injection of [^{18}F]FPy-BCMA-Nb.

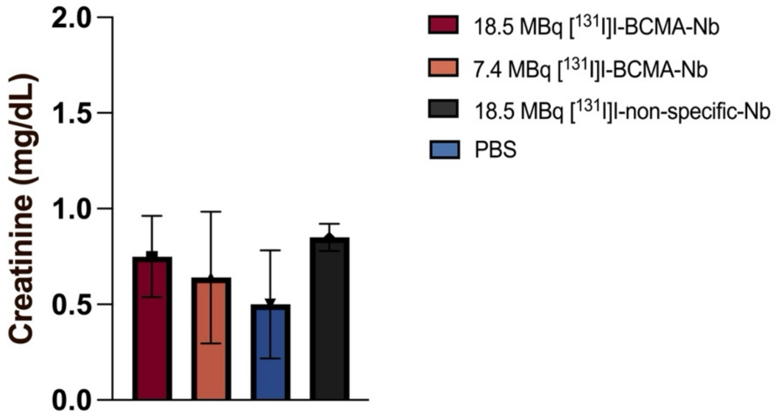

Supplemental Figure S12. Serum creatinine levels measured in all mice after completion of treatment across different groups. No significant differences were observed between groups, indicating the absence of treatment-related nephrotoxicity.

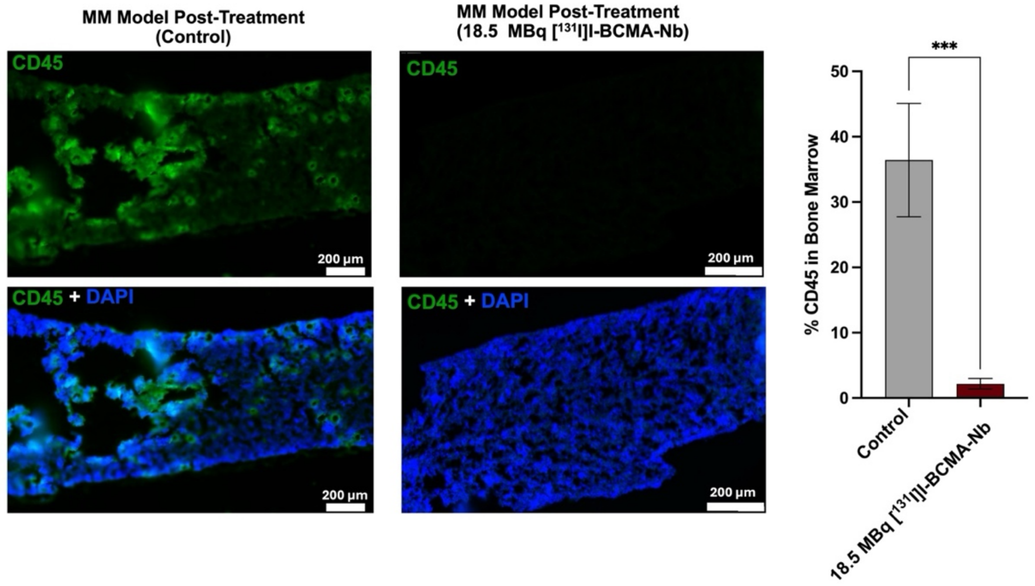

Supplemental Figure S13. Immunohistochemistry staining and corresponding quantitative analysis of CD45 in femoral bone marrow sections from H929 human multiple myeloma–bearing mice in the control group and after treatment with 18.5 MBq [^{131}I]I-BCMA-Nb. *** indicates $p < 0.001$.

Supplemental Table S1. Complete blood count (CBC) values measured before treatment across all groups. Data are presented as mean ± SD.

	Pre-treatment				
	PBS	7.4 MBq [^{131}I]I-BCMA-Nb	18.5 MBq [^{131}I]I-BCMA-Nb	18.5 MBq [^{131}I]I -non-specific-Nb	Unit
**WBC Count**	1.56 ± 0.4	1.06 ± 0.3	1.56 ± 0.3	1.81 ± 0.4	K/µL
**RBC Count**	10 ± 1.8	10 ± 1.5	10.93 ± 1.7	10.81 ± 2.1	M/µL
**Hemoglobin**	13.12 ± 0.8	13.75 ± 1.2	13.12 ± 0.8	13.75 ± 0.9	g/dL
**Hematocrit**	44.43 ± 2.7	44.37 ± 3.1	48.56 ± 2.8	47.25 ± 3.1	%
**MCV**	44.75 ± 2.2	44.5 ± 0.5	44.25 ± 0.7	43.5 ± 1.2	fL
**Platelet**	675	793	637.5	600	K/µL
